# Within the Ischemic Penumbra, Sub-Cellular Compartmentalization of Heat Shock Protein 70 Overlaps with Autophagy Proteins and Fails to Merge with Lysosomes

**DOI:** 10.3390/molecules27103122

**Published:** 2022-05-13

**Authors:** Federica Mastroiacovo, Francesca Biagioni, Paola Lenzi, Gloria Lazzeri, Michela Ferrucci, Stefano Puglisi-Allegra, Alessandro Frati, Ferdinando Nicoletti, Francesco Fornai

**Affiliations:** 1 I.R.C.C.S. Neuromed, Via Atinense 18, 86077 Pozzilli, Italy; federica.mast@neuromed.it (F.M.); francesca.biagioni@neuromed.it (F.B.); stefano.puglisiallegra@neuromed.it (S.P.-A.); alessandro.frati@uniroma1.it (A.F.); nicoletti@neuromed.it (F.N.); 2 Department of Translational Research and New Technologies in Medicine and Surgery, University of Pisa, Via Roma 55, 56126 Pisa, Italy; paola.lenzi@unipi.it (P.L.); gloria.lazzeri@unipi.it (G.L.); michela.ferrucci@unipi.it (M.F.); 3 Neurosurgery Division, Department of Human Neurosciences, Sapienza University, 00135 Rome, Italy; 4 Department of Physiology and Pharmacology, University Sapienza of Rome, 00135 Rome, Italy

**Keywords:** autophagy vacuoles, lysosomes, cell clearing systems, ultrastructural stoichiometry

## Abstract

The brain area which surrounds the frankly ischemic region is named the *area penumbra*. In this area, most cells are spared although their oxidative metabolism is impaired. *area penumbra* is routinely detected by immunostaining of a molecule named Heat Shock Protein 70 (HSP70). Within the *area penumbra*, autophagy-related proteins also increase. Therefore, in the present study, the autophagy-related microtubule-associated protein I/II-Light Chain 3 (LC3) was investigated within the *area penumbra* along with HSP70. In C57 black mice, ischemia was induced by permanent occlusion of the distal part of the middle cerebral artery. Immunofluorescence and electron microscopy show that LC3 and HSP70 are overexpressed and co-localize within the *area penumbra* in the same cells and within similar subcellular compartments. In the *area penumbra*, marked loss of co-localization of HSP70 and LC3-positive autophagy vacuoles, with lysosomal-associated membrane protein 1 (LAMP1) or cathepsin-D-positive lysosome vacuoles occurs. This study indicates that, within the *area penumbra*, a failure of autophagolysosomes depends on defective compartmentalization of LC3, LAMP1 and cathepsin-D and a defect in merging between autophagosomes and lysosomes. Such a deleterious effect is likely to induce a depletion of autophagolysosomes and cell clearing systems, which needs to be rescued in the process of improving neuronal survival.

## 1. Introduction

**Figure 1:**
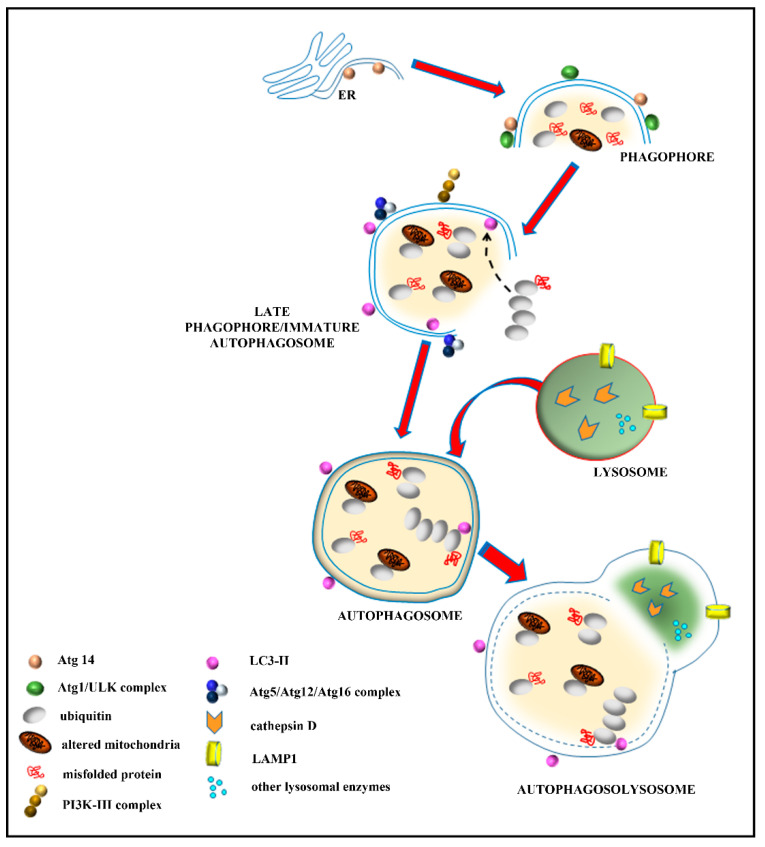
The autophagy machinery. Autophagy is initiated by the autophagy initiation complex, named Atg1 or Unc51-like autophagy activating kinase (ULK) complex. Atg1/ULK complex induces the formation of an incomplete cup-shaped double-membrane structure, called phagophore from the mitochondrial and/or endoplasmic reticulum (ER) membranes. Phosphorylation of specific proteins of the phagophore membrane (such as Atg14) by the Atg1/ULK complex allows the activation of beclin 1 and its binding to phosphatidylinositol-3-kinase class III (PI3K-III)/VPS34. This leads to the formation of the active PI3K-III complex, which produces a focal increase in phosphatidylinositol-3-phosphate (PI3P), followed by a variety of reactions, leading to the recruitment of LC3. LC3 (microtubule-associated protein I/II-Light Chain 3), an Atg8 family member, is cleaved by Atg4 to form LC3-I in the cytosol; then, cytoplasmic LC3-I is covalently bound to phosphatidylethanolamine on the phagophore membrane to form LC3-II. This latter reaction, called LC3 lipidation, is induced by the Atg5/Atg12/Atg16 complex. These events occur in the late phagophore/immature autophagosome, which folds and enwraps the damaged cell components, such as ubiquitinated mitochondria and ubiquitinated proteins, in the autophagosome. During its progression, the autophagosome is driven along microtubules to merge with LAMP1- and cathepsin-D-positive lysosomes to constitute the autophagolysosome, where a variety of hydrolases are responsible for cargo degradation.

**Figure 2:**
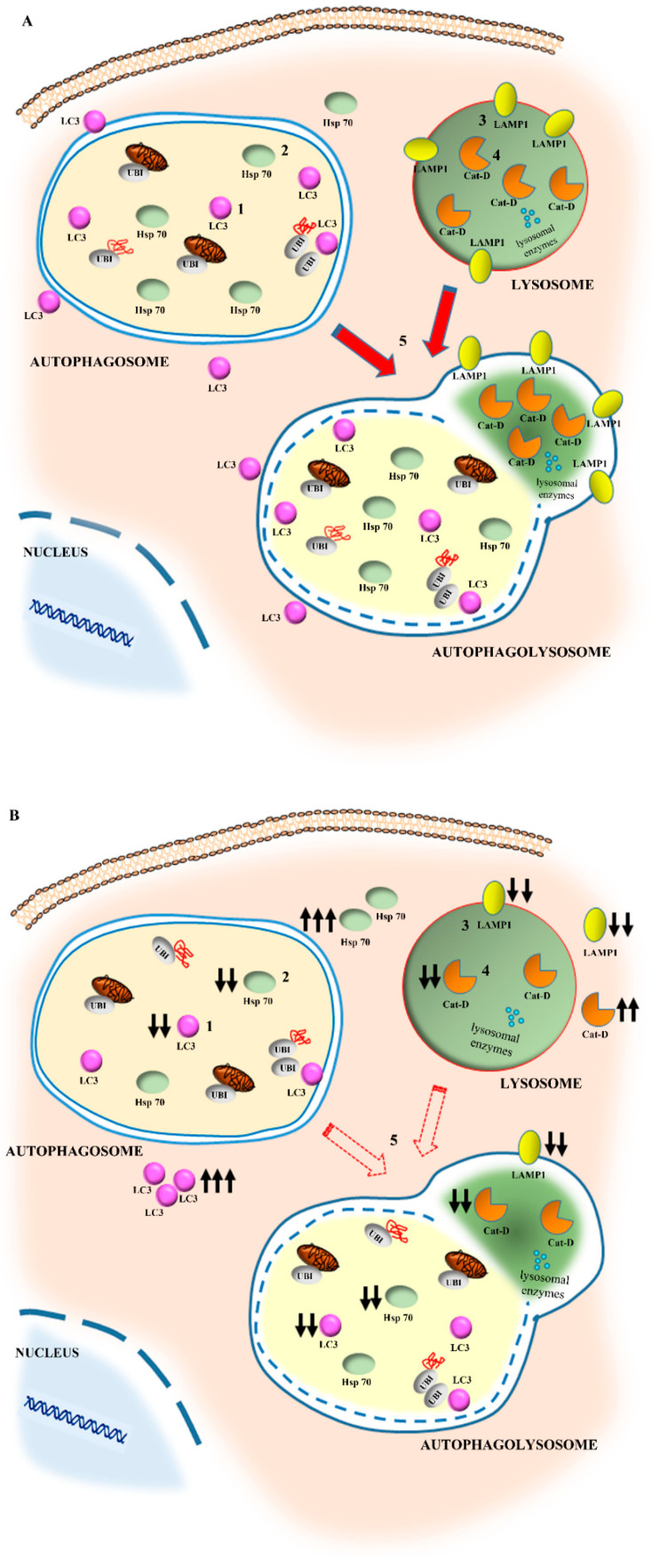
Compartmentalization of specific autophagy molecules in physiological conditions (**A**) and within the *area penumbra* neurons (**B**). In detail, in control conditions (**A**), LC3 (**1**), which stains the autophagosomes, co-localizes with HSP70 (**2**) within the same vacuoles. In ischemia (**B**), there is a loss of both LC3 (**1**) and HSP70 (**2**) in the vacuoles as shown by the downward arrows, while both molecules greatly increase within cytosol (as shown by triple upward arrows in B). In the lysosome compartment both LAMP1 (**3**) and cathepsin-D (CAT D) (**4**) can be found in control conditions (**A**). During ischemia (**B**), there is a loss of LAMP1 (**3**) both in vacuoles (double downward arrows) and cytosol (double downward arrows). In ischemia (**B**), cathepsin-D (**4**) is reduced within vacuoles (double downward arrow) while it markedly increases in the cytosol (double upward arrows). In these conditions, the co-localization of the autophagosome marker LC3 (**1**) with the lysosome marker LAMP1 (**3**) within vacuoles is suppressed. This occurs more markedly than the suppression of each molecule from autophagosome and lysosome, respectively. This suggests that the massive defect in autophagolysosome formation is also due to a decrease in merging between vacuoles (**5**).

A number of data collected over the last few decades define the ischemic *penumbra* as a critical region identified by magnetic resonance imaging (MRI). This region represents the border between ischemia-induced frank cell loss (ischemic core) and healthy brain tissue. Thus, the *area penumbra* corresponds to a peri-infarct region [1,2,3]. Within the *area penumbra*, the nervous matter, mainly neurons, are on the edge concerning cell viability. In fact, within this area cells undergo either delayed, post-ischemic cell death or recovery, depending on which ongoing pharmacological and/or physiological event is taking place [4]. In recent decades, the *area penumbra* was intensely studied in an effort to improve post-ischemic maturation phenomena [4]. At the biochemical level, the *area penumbra* carries a defect of mitochondrial oxidative metabolism. In pre-clinical studies, the *area penumbra* is routinely identified as a region overexpressing the chaperone Heat Shock Protein 70 (HSP70) [5,6,7]. Within the *area penumbra* neurons, the amount of HSP70 increases in the whole cell, although its presence within vacuoles is reduced (Figure 1 and Figure 2) [3].

Recent studies indicate that, within the *area penumbra*, autophagy-related proteins are consistently overexpressed [2,8,9,10]. This increase is best described for the autophagy protein microtubule-associated protein I/II-Light Chain 3 (LC3). The significance of such an overexpression is reported to be either deleterious [11] or protective [12,13]. In fact, the role of autophagy-related proteins remains under debate [14]. The increase in the autophagy-related protein LC3 was established in the elegant studies by the Rami’s team [8,9,10]. This indicates that LC3 may be considered as a marker of the *area penumbra* as much as HSP70. However, no study so far compared, in the same experimental settings, the increase in LC3 in combination with HSP70.

Therefore, the present study analyzes the co-expression of HSP70 and LC3 within the penumbra region, its cells and its specific cell compartments. This is carried out following persistent ischemia, which was experimentally produced by occluding the distal middle cerebral artery (MCAO). To measure concomitant expression of HSP70 and LC3, immunofluorescence was joined with stoichiometric measurements of each molecule by transmission electron microscopy (TEM). The measurement of sub-cellular placement of LC3 in combination with the marker HSP70 allows us to establish whether these proteins share similar sub-cellular compartments, where they may synergize. In this effort, autophagy vacuoles need to be primarily analyzed. In fact, previous studies show that up-regulation of cytosol LC3 within ischemic penumbra copes with a failure of LC3 to merge with lysosomal compartment [15]. This was originally attributed to stagnant autophagosomes, which may accumulate in excess compared with the ability of lysosomes to clear these vacuoles [8]. In the present study, the merging of both HSP70 and LC3 with the lysosomal-associated membrane protein 1 (LAMP1) and cathepsin-D is analyzed (Figure 1 and Figure 2). This allows us to establish whether an excess of autophagosomes does occur in the *area penumbra*. Similarly, the occurrence of lysosome-specific markers was analyzed. This allows us to establish whether a primary failure of both autophagosome- and lysosome-competent proteins occurs. This is questioned by scoring immunofluorescence at light microscopy and by counting each authentic molecule in situ, within its specific organelle. Again, the merging between markers specific for autophagosomes and lysosomes allows us to answer the question as to whether merging between these compartments is altered. Moreover, assessing the placement of HSP70 within autophagosome and its merging with lysosome extends our vision about specific roles played by HSP70 as a chaperone within specific compartments of ischemic neurons. In fact, the merging of LC3 and HSP70 with LAMP1 and cathepsin-D indicates a potential common fate of these molecules, which mark the *area penumbra*.

## 2. Results

### 2.1. Outcome of Middle Cerebral Artery Occlusion (MCAO)

**Figure 3:**
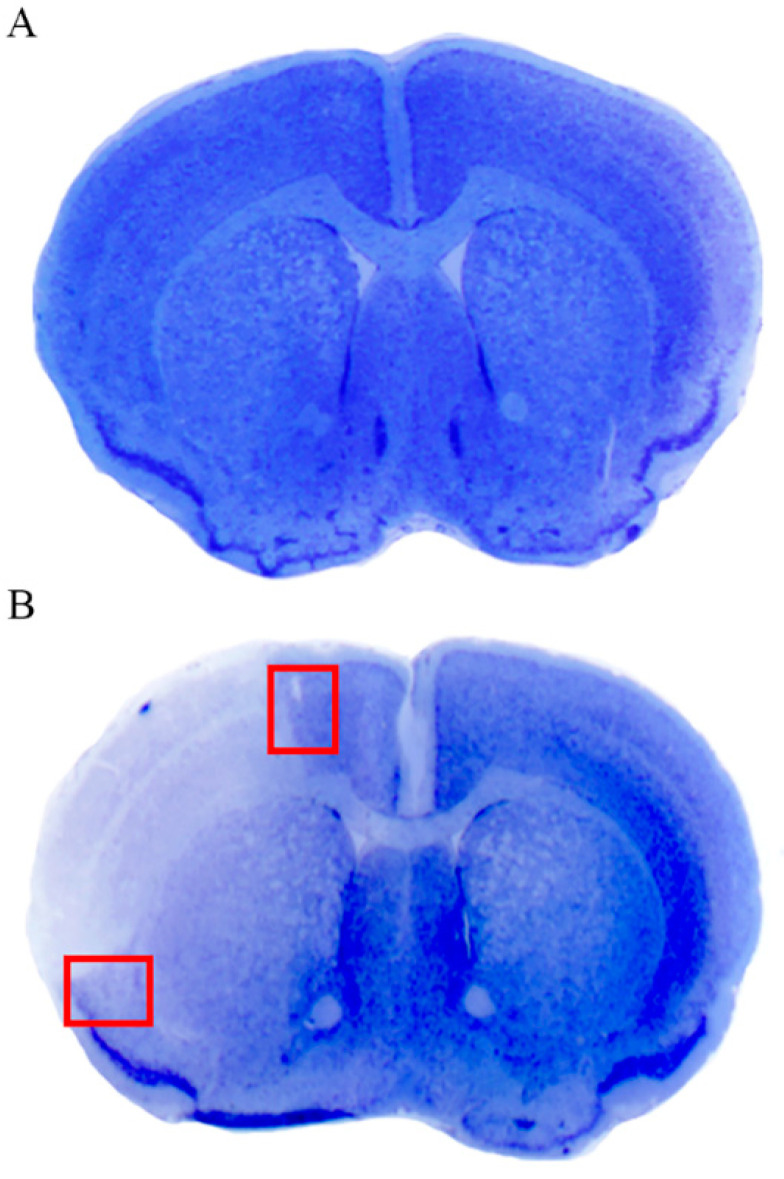
Thionin staining shows a pale ischemic brain area following middle cerebral artery occlusion (MCAO). (**A**) shows representative thionin staining from a sham-operated mouse, following the surgical procedure without occlusion of the middle cerebral artery (sham-operated, sham). (**B**) is a representative thionin staining from a mouse following right MCAO. The pale cortical and sub-cortical areas represent ischemic regions. In (**B**), red squares indicate the dorsal and ventral peri-infarct regions, which were selected for anatomical studies of the *area penumbra*.

Following distal (Sylvian) occlusion of the middle cerebral artery (MCA), a wide permanent ischemic damage is produced in the ipsilateral hemisphere. This involves both cortical and sub-cortical regions (including the white matter and basal ganglia). As shown in Figure 3, the cortical peri-infarct area is evident following thionin staining as a region, where a sudden chromatic shift occurs from the abnormal pale necrotic tissue to the normal blue Nissl-stained brain matter. The cortical infarct area is well characterized as the pale region delimited by a dorsal and ventral peri-infarct area. The present study focuses on the peri-infarct region, as the presumed site of the *area penumbra*. Therefore, the cortical areas, which are selected for the anatomical study, correspond to those delimited by red squares of Figure 3. These can be distinguished into a ventral (basal) region and a dorsal (apical) region (Figure 3B). Some differences between dorsal and ventral regions were recently noticed [2]. These are likely to rely on a different vulnerability to ischemia of ventral allo-cortical (more damaged) compared with dorsal iso-cortical (less damaged) neurons [2]. Nonetheless, changes produced by ischemic damage are qualitatively similar within the dorsal and ventral regions. Thus, data from the ventral areas are included in the main body of the text, while data from the dorsal areas (largely confirmatory) are reported in the Supplementary Figures S2–S6.

Ischemic damage extends rostro-caudally from the rostral pole of the striatum to the anterior thalamic nuclei, while the cortical reference spans from the primary motor cortex (M1) to the auditory cortex. Within this region, the red squared areas were dissected both ventrally and dorsally to obtain small blocks from analogous regions in the ischemic side, contralateral side and from homologous areas of sham-operated mice.

**Figure 4:**
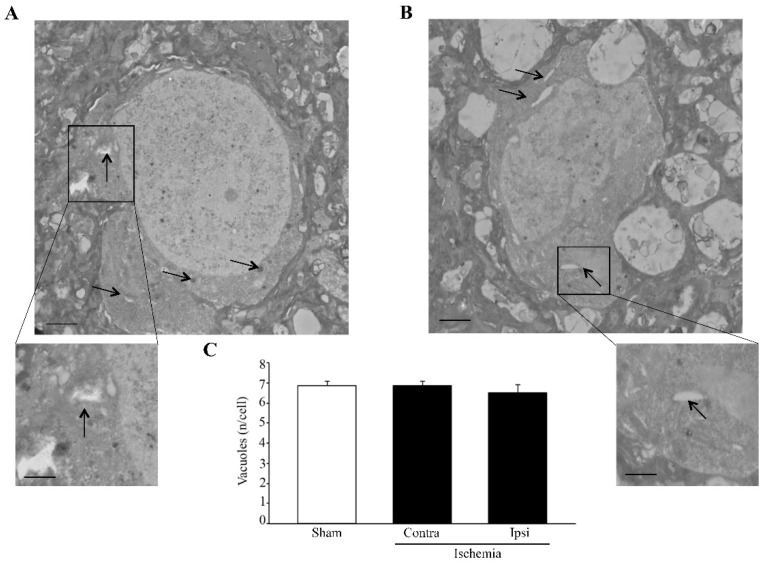
Within the *area penumbra*, non-stained vacuoles are evident and do not change compared with the control. (**A**) shows representative transmission electron microscopy (TEM) micrograph from a cortical neuron of the ventral region homologous to the site of the *area penumbra* from a sham-operated mouse (sham). (**B**) shows a micrograph from a cortical neuron from the ventral *area penumbra* from an ischemic mouse. The representative pictures allow us to distinguish non-stained vacuoles (arrows). Each insert reports vacuoles at higher magnification. Graph (**C**) reports the number of vacuoles within the *area penumbra* (Ipsi/Ischemia) and homologous regions from the contralateral side (Contra/Ischemia) and from sham-operated mice (Sham). Values are given as the mean ± S.E.M. per cell from 120 cells counted in each group. Scale bar = 0.2 µm (**A**,**B**), 0.1 µm (inserts).

Sub-cellular structures such as vacuoles are evident in each group (Figure 4). Direct visualization of the *area penumbra* neurons compared with non-ischemic neurons is reported in Figure 4. The TEM procedure allows both the identification and counting of small cell compartments, such as vacuoles. Remarkably, as reported in the graph of Figure 4C, within *area penumbra*, the number of non-stained vacuoles is not modified. It is rather the specific staining which is modified, as we shall see.

### 2.2. Effects of MCAO on LC3 and HSP70 Immunofluorescence within the area penumbra

**Figure 5:**
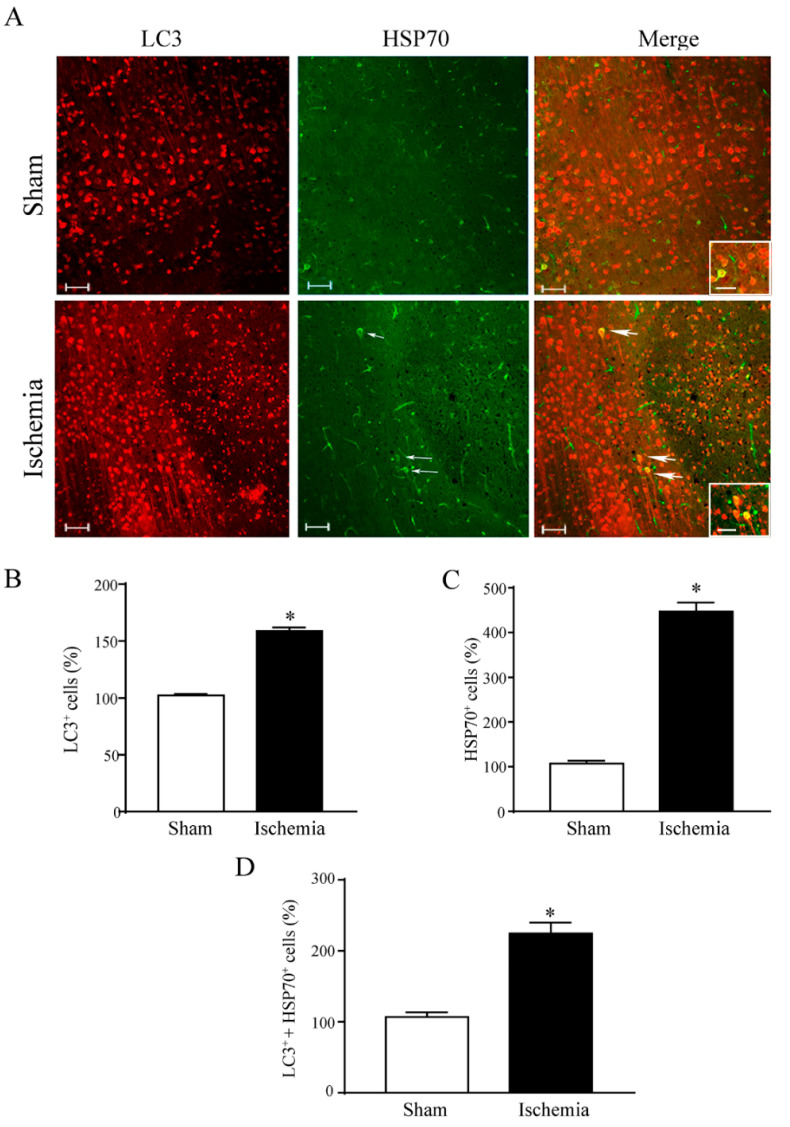
Within the *area penumbra*, Heat Shock Protein 70 (HSP70) co-localizes with microtubule-associated protein I/II-Light Chain 3 (LC3). (**A**) shows representative pictures of LC3 immunofluorescence (red), HSP70 immunofluorescence (green) and merging these antigens. Graph (**B**) reports LC3-positive cells within the *area penumbra* (Ischemia) compared with the control (Sham). Graph (**C**) reports HSP70-positive cells within the *area penumbra* (Ischemia) compared with the control (Sham). Arrows point the merging of LC3 with HSP70. Graph (**D**) reports LC3- + HSP70-positive cells in the ischemic *area penumbra* (Ischemia) compared with the control (Sham). Values are reported as percentage of control and are given as the mean ± S.E.M. of 12 values for each group (n = 3 slices × N = 4 mice). * *p* ≤ 0.05 compared with other groups. Scale bars = 50 µm; 10 µm (inserts) (**A**).

As shown in representative Figure 5A, LC3 immunofluorescence stains the *area penumbra* similarly to HSP70 immunofluorescence, which is the classic marker of the *area penumbra*. The present investigation provides the first evidence for co-expression of both antigens within the *area penumbra*. In detail, as reported in the graphs of Figure 5B, LC3-positive cells increase in the *area penumbra* compared with analogous regions from sham-operated mice. Such an increase mimics what occurs for HSP70 (Figure 5C). When counted following single staining, the increase in HSP70 (Figure 5C) exceeds the increase in LC3 (Figure 5B). When merging LC3 and HSP70 immunofluorescence, the difference between baseline and ischemic condition is enhanced even compared with HSP70 staining alone as reported in the graph of Figure 5D. This indicates that combining both antigens provides a more sensitive marker to detect the *area penumbra*. Again, the marked increase in co-localization measured in the graph of Figure 5D indicates that HSP70 and LC3 mark the same neurons within the *area penumbra*.

### 2.3. Effects of MCAO on HSP70 and LC3 Immuno-Gold Stoichiometry within the area penumbra

**Figure 6:**
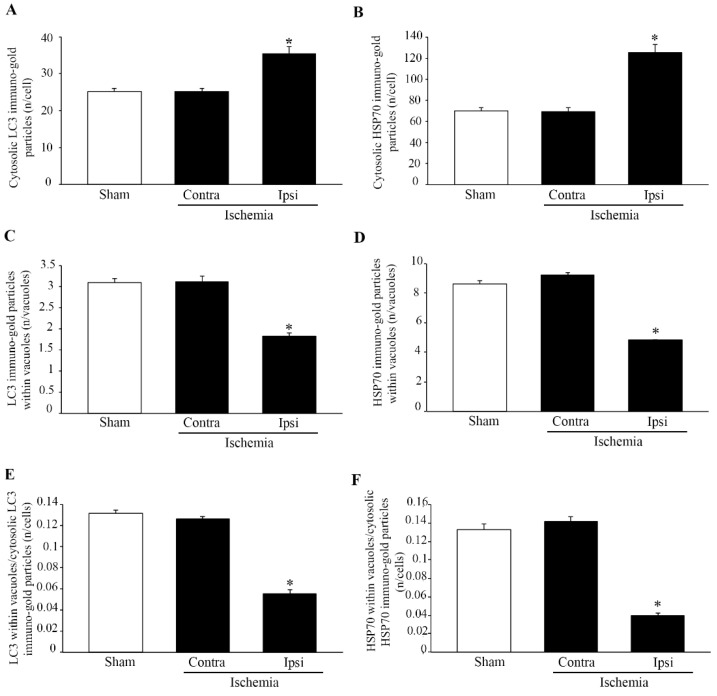
Within the *area penumbra*, Heat Shock Protein 70 (HSP70) and microtubule-associated protein I/II-Light Chain 3 (LC3) shift from vacuole to cytosol. Graphs on the left side report the amount of LC3 particles within cytosol (**A**), within vacuoles (**C**) and the ratio between vacuolar and cytosolic LC3 particles (**E**). Similarly, the graph on the right side reports the amount of HSP70 particles within cytosol (**B**), within vacuoles (**D**) and the ratio between vacuolar and cytosolic HSP70 particles (**F**). Particles are counted in sham-operated mice (Sham), on the side contralateral to ischemia (Contra/Ischemia) and within the *area penumbra* (Ipsi/Ischemia). Values are reported as the mean ± S.E.M. of immuno-gold particle counted in each cell from a total of 120 cells for each group. * *p* ≤ 0.05 compared with other groups.

A detailed assessment of the amount and placement of HSP70 and LC3 at ultrastructural morphometry is reported in the graphs of Figure 6. These graphs report an increase in the number of LC3 molecules counted in the cytosol of the *area penumbra* neurons (Figure 6A). The increase in HSP70 molecules, which similarly occurs in the *area penumbra* neurons, is more robust compared with LC3 (Figure 6B). On the contrary, when the very same molecules were counted within vacuoles of the *area penumbra* neurons, a significant decrease in both LC3 and HSP70 is detected in the ischemic compared with the contralateral side and both sides from sham-operated mice (graph of Figure 6C,D, respectively). The shifting in vacuolar vs. cytosolic compartmentalization of LC3 and HSP70 confirms what has been recently reported [3].

The shifting in the cytosolic vs. vacuolar compartmentalization of LC3 is slighter compared with the massive effects detected for the ratio of vacuolar vs. cytosolic HSP70 (Figure 6E,F, respectively).

**Figure 7:**
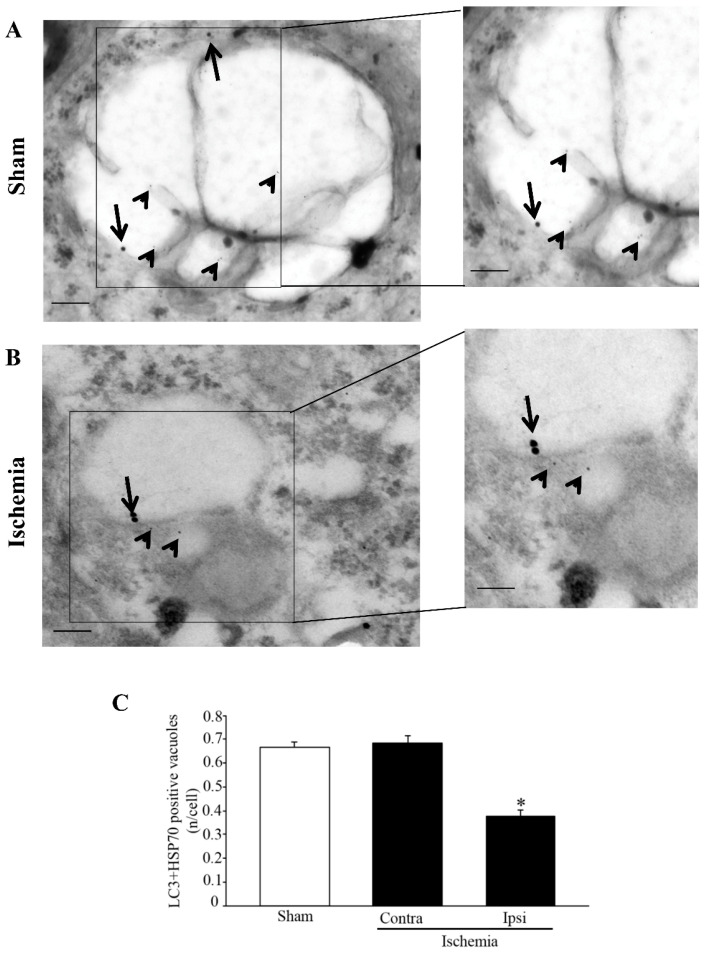
Microtubule-associated protein I/II-Light Chain 3 (LC3) + Heat Shock Protein 70 (HSP70) co-immuno-stained vacuoles decrease within the *area penumbra*. (**A**) shows representative TEM micrographs of LC3 + HSP70 co-immuno-stained vacuoles within cortical neuron from a region homologous to the *area penumbra* from a sham-operated mouse (Sham). (**B**) shows a cortical neuron from the *area penumbra* from an ischemic mouse (Ischemia). Arrows point to LC3 (20 nm) and arrowheads point to HSP70 (10 nm) immuno-gold particles within vacuoles. The insert report LC3 + HSP70 immuno-gold particles within vacuoles at higher magnification. Graph (**C**) reports the number of LC3 + HSP70 immuno-gold double-stained vacuoles per cell from sham-operated mice (Sham), from homologous regions contralateral to the ischemic side (Contra/Ischemia) and from the *area penumbra* (Ipsi/Ischemia). Values are given as the mean ± S.E.M. per cell counted in 120 cells in each group. * *p* ≤ 0.05 compared with other groups. Scale bar = 0.2 µm (**A**,**B**), 0.1 µm (inserts).

The vacuolar co-localization of LC3 and HSP70 is reported in the representative pictures of Figure 7. In detail, LC3 + HSP70 immuno-gold particles are shown in the neurons from a sham mouse (Figure 7A) and from the *area penumbra* of an ischemic mouse (Figure 7B).

The graph of Figure 7C indicates that, analogous to single antigen detection, even the amount of LC3 and HSP70 co-localized within the very same vacuoles is suppressed in neurons from the *area penumbra* compared with the contralateral side and analogous regions from sham-operated mice.

Altogether, these findings indicate that LC3 and HSP70 both provide a zonal staining of the *area penumbra*, being increased within the same neurons, although being both dissipated from vacuoles to cytosol in ischemic conditions. The vacuolar compartment under analysis corresponds to autophagosomes. In fact, it consists of LC3-positive multiple membrane vacuoles owing a similar electron density compared with surrounding cytosol. Thus, the present findings provide additional information concerning the amount of autophagy vacuoles within *area penumbra* neurons and the placement of HSP70 within autophagy vacuoles in the very same neurons of the *area penumbra*. In order to better detail the relationship between LC3 and HSP70 within the same vacuoles, we measured the mean distance between these molecules when co-localized. No differences were found in sham-operated (701.9 ± 0.048 nm) and in homologous regions contralateral to the *area penumbra* (694.4 ± 0.054 nm) compared with the *area penumbra* (683.2 ± 0.049 nm). The range of variability (95%) spans from 200 nm to 1520 nm. The distance is homogenously distributed, with no clustering of co-localization being detectable at any distance. In contrast, when an interaction is expected (as in the case of aggregates of LC3 molecules) stoichiometry reveals a distance approaching zero (when immuno-gold particles are grouped (see representative images of Figure 7). The effects of ischemia similarly affect the compartmentalization of LC3 and HSP70. In fact, within the *area penumbra*, the mean number of LC3 molecules within each autophagy vacuoles is decreased similarly to the decrease in HSP70 molecules within the very same autophagy vacuoles. This is a fundamental piece of evidence since previous studies hypothesized that the up-regulation of LC3 within the whole cell of the *area penumbra* may be due to stagnant autophagosomes, which do not merge with lysosomes [8,9,15,16,17]. This was inferred based on the interpretation of immunofluorescence, which did not allow us to dissect the non-vacuolar placement of LC3 and led to interpret cytosolic LC3 aggregates as big vacuoles. Instead, present findings indicate that a primary defect in LC3-positive autophagosomes occurs in *area penumbra* neurons, although a whole cell increase in LC3 is present. Thus, a failure of LC3 to merge with the lysosomal compartment is due, at least in part, to a primary failure of LC3 to compartmentalize within autophagosomes.

To explore this issue more in depth, the present study analyzes the fate of LC3 in relationship with the lysosome vacuoles. This was carried out to question whether a concomitant failure of LC3-positive autophagosomes to merge with lysosomes is present. To this aim, the status of lysosome vacuoles and lysosome-competent proteins, such as LAMP1 and cathepsin-D, were also assessed.

### 2.4. Effects of MCAO on LC3 and LAMP1 Immunofluorescence within the area penumbra

**Figure 8:**
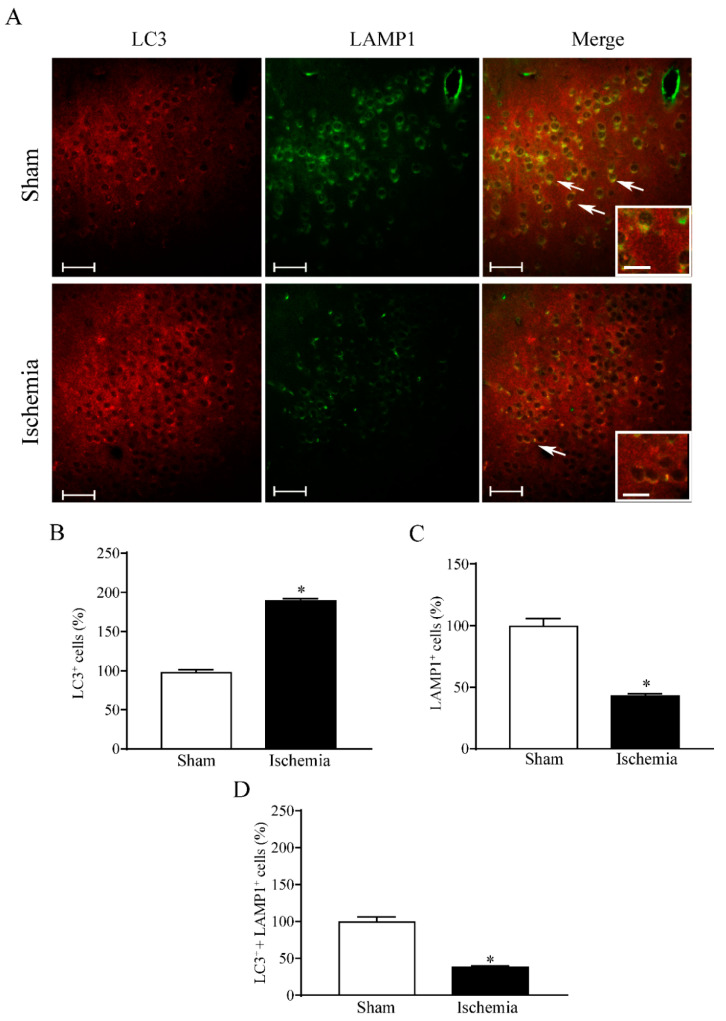
Within the *area penumbra*, microtubule-associated protein I/II-Light Chain 3 (LC3) + lysosomal-associated membrane protein 1 (LAMP1) co-localization is suppressed. (**A**) shows representative pictures of LC3 immunofluorescence (red), LAMP1 immunofluorescence (green) and merge of both antigens. Arrows point to the merge of LC3 and LAMP1. Graph (**B**) reports the increase in LC3-positive cells within the *area penumbra* (Ischemia) compared with the control (Sham). Graph (**C**) reports the decrease in LAMP1-positive cells within the *area penumbra* (Ischemia) compared with the control (Sham). Graph (**D**) reports the suppression of LC3- + LAMP1-positive cells within the ischemic *area penumbra* (Ischemia) compared with the control (Sham). Values are reported as percentage of control and are given as the mean ± S.E.M. of 12 values for each group (n = 3 slices × N = 4 mice). * *p* ≤ 0.05 compared with other groups. Scale bars = 50 µm; 10 µm (inserts) (**A**).

The increase in the percentage of LC3-positive cells within the *area penumbra* is confirmed in Figure 8A and it is measured in the graph of Figure 8B. In contrast, the lysosome-related antigen LAMP1 is markedly suppressed within whole cells from the *area penumbra* (Figure 8C). Such a decrease is exacerbated when the double staining (merge) for LC3 and LAMP1 is reported in the graph of Figure 8D. In fact, within the *area penumbra*, the percentage of cells where LAMP1 and LC3 merge is dramatically reduced. Such a decrease results in multiple additional defects concerning autophagolysosomes. In fact, a primary defect in the cellular amount of LAMP1 further impairs the lysosomal compartment and its merging with autophagosomes. This adds on the specific loss of LC3 from vacuoles in *area penumbra* neurons.

These findings suggest that a failure of the lysosome-related clearance within the *area penumbra* occurs as a consequence of a loss of competence of the lysosome compartment rather than an over-accumulation of autophagosomes. In fact, as previously reported in Figure 6, the number of LC3 within autophagosomes is reduced within the *area penumbra*. In line with this, the number of non-stained vacuoles remain steady within the *area penumbra* (Figure 4C) and the same happens for the number of HSP70-positive vacuoles (Figure S1A). In summary, these data indicate that LC3-positive autophagy vacuoles markedly decrease within the *area penumbra* as reported in Supplementary Figure S1B.

### 2.5. Effects of MCAO on LAMP1 Immuno-Gold Stoichiometry within the area penumbra

**Figure 9:**
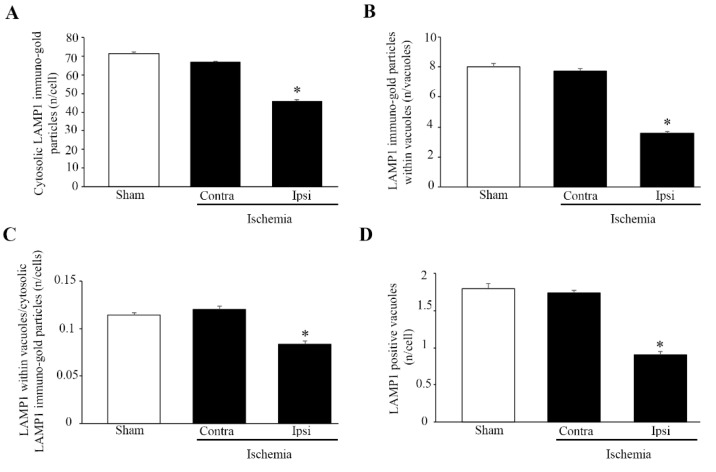
Within *area penumbra* cells, lysosomal-associated membrane protein 1 (LAMP1) molecules are reduced and dissipated from vacuoles. Graph (**A**) reports a reduced amount of LAMP1 molecules counted in the cytosol of neurons from the *area penumbra* (Ipsi/Ischemia) compared with the amount measured within neurons from homologous regions of the contralateral side (Contra/Ischemia) and from sham-operated mice (Sham). Graph (**B**) reports a massive decrease in the amount LAMP1 molecules counted within vacuoles of neurons from the *area penumbra* (Ipsi/Ischemia) compared with the amount measured within vacuoles from neurons from homologous regions of the contralateral side (Contra/Ischemia) and sham-operated mice (Sham). Graph (**C**) reports a decrease in the ratio between vacuolar and cytosolic amount of LAMP1 molecules within neurons from the *area penumbra* (Ipsi/Ischemia) compared with the ratio measured within neurons from homologous regions of the contralateral side (Contra/Ischemia) and from sham-operated mice (Sham). Graph (**D**) reports the decrease in LAMP1-positive vacuoles, which are counted within neurons from the *area penumbra* (Ipsi/Ischemia) compared with the amount measured within neurons from homologous regions of the contralateral side (Contra/Ischemia) and sham-operated mice (Sham). Values are reported as the mean ± S.E.M. per cell counted in 120 cells for each group. * *p* ≤ 0.05 compared with other groups.

In order to validate light microscopy data and quantify the amount of LAMP1 molecules at ultrastructural morphometry, the stoichiometry count of LAMP1 was carried out in the cytosol of neurons from the *area penumbra* compared with analogous regions from the contralateral side and sham-operated mice. Similar to immunofluorescence, the count of LAMP1 molecules in the cytosol decreases within the *area penumbra* (Figure 9A). Such a decrease is more marked when LAMP1 immuno-gold particles are counted within vacuoles from the *area penumbra* (Figure 9B). As expected from a loss of compartmentalization, the decrease in LAMP1 within vacuoles is more evident when compared with the decrease which was counted in whole cytosol. This is consistent with a decrease in the ratio between LAMP1 immuno-gold particles within vacuoles and cytosol, which was counted in the *area penumbra* (Figure 9C). This indicates that, despite being decreased in both cytosol and vacuoles, the loss of LAMP1 mostly affects the vacuolar compartment. This is consistent with a massive decrease in the number of LAMP1-positive vacuoles in the *area penumbra* (Figure 9D). Such a decrease recapitulates the reduction counted in Figure 9B for LAMP1 immuno-gold particles within vacuoles. This strengthens the evidence indicating a primary depletion of active molecules from lysosome compartment, which is concomitant with a loss in the number of lysosome-like vacuoles.

**Figure 10:**
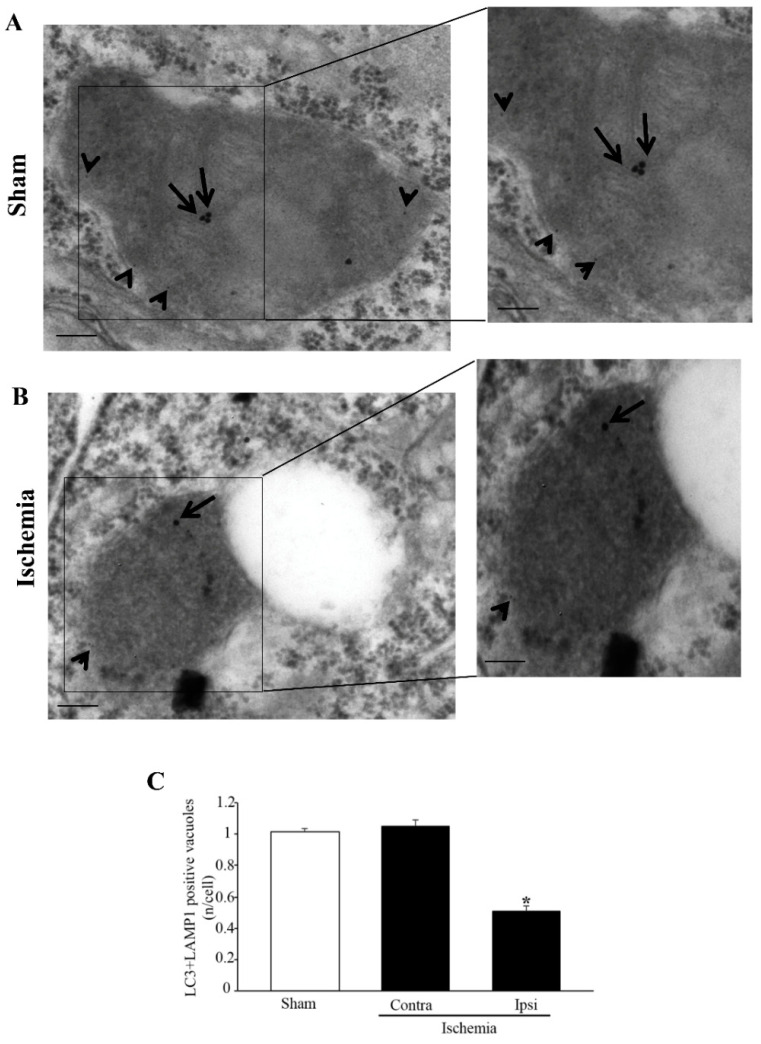
Microtubule-associated protein I/II-Light Chain 3 (LC3) + lysosomal-associated membrane protein 1 (LAMP1) co-immuno-stained vacuoles decrease within the *area penumbra*. (**A**) shows representative TEM micrographs showing LC3 + LAMP1 co-immuno-stained vacuoles within a cortical neuron from a region homologous to the *area penumbra* from a sham-operated mouse (Sham), while (**B**) shows a cortical neuron from the *area penumbra* from an ischemic mouse (Ischemia). Arrows point to LC3 (20 nm) and arrowheads point to LAMP1 (10 nm) immuno-gold particles within vacuoles. The insert reports LC3 + LAMP1 immuno-gold particles within vacuoles at higher magnification. Graph (**C**) reports the number of LC3 + LAMP1 immuno-gold double-stained vacuoles within cells from the *area penumbra* (Ipsi/Ischemia) and homologous regions from the contralateral side (Contra/Ischemia) and sham-operated mice (Sham). Values are given as the mean ± S.E.M. per cell counted in 120 cells in each group. * *p* ≤ 0.05 compared with other groups. Scale bar = 0.2 µm (**A**,**B**), 0.1 µm (inserts).

In order to document the merging between autophagosomes and lysosomes (through their markers LC3 and LAMP1, respectively, within vacuoles) double-immuno-gold stoichiometry was carried out. This is shown in representative pictures of Figure 10 either from a sham-operated (Figure 10A) or an ischemic mouse (Figure 10B). As reported in the graph of Figure 10C, a decrease in LAMP1- and LC3-positive vacuoles occurs within the *area penumbra*. This recapitulates the decrease in LAMP1-positive vacuoles reported in the graph of Figure 9D. This suggests that a massive failure of LAMP1-positive lysosomal compartment characterizes the *area penumbra*.

### 2.6. Effects of MCAO on Cathepsin-D Immuno-Gold Stoichiometry within the area penumbra

**Figure 11:**
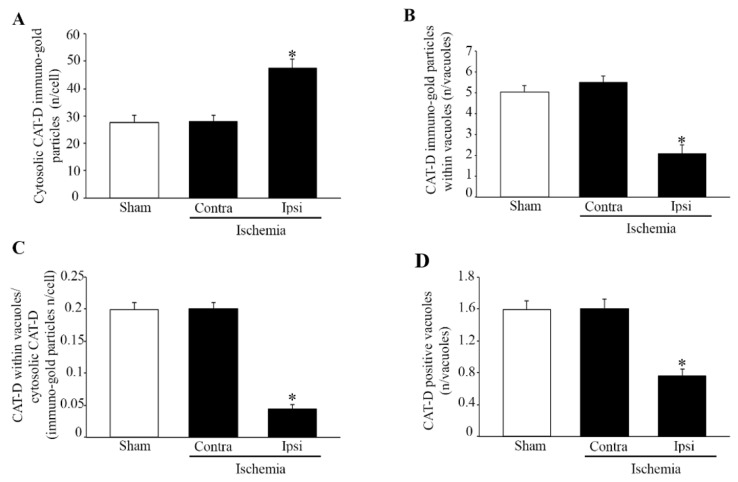
Within the *area penumbra*, increased cathepsin-D (CAT-D) molecules are dispersed in the cytosol. Graph (**A**) reports an increase in CAT-D molecules in the cytosol within neurons from the *area penumbra* (Ipsi/Ischemia) compared with homologous regions from the contralateral side (Contra/Ischemia) and from sham-operated mice (Sham). Graph (**B**) reports a decrease in the amount of CAT-D molecules within vacuoles within *area penumbra* neurons from (Ipsi/Ischemia) compared with homologous regions from the contralateral side (Contra/Ischemia) and sham-operated mice (Sham). Graph (**C**) reports a massive decrease in the ratio between cytosolic and vacuolar CAT-D molecules within neurons from the *area penumbra* (Ipsi/Ischemia) compared with homologous regions from the contralateral side (Contra/Ischemia) and sham-operated mice (Sham). Graph (**D**) reports a massive decrease in the amount of CAT-D-positive vacuoles counted within neurons from the *area penumbra* (Ipsi/Ischemia) compared with homologous regions from the contralateral side (Contra/Ischemia) and sham-operated mice (Sham). Values are reported as the mean ± S.E.M. per cell counted in 120 cells from each group. * *p* ≤ 0.05 compared with other groups. CAT-D = cathepsin-D.

In order to strengthen the evidence showing a failure of the lysosome compartment within the *area penumbra*, the amount and placement of cathepsin-D molecules were quantified at ultrastructural morphometry. The stoichiometry count of cathepsin-D occurring in the cytosol and vacuoles of neurons from the *area penumbra* was compared with analogous regions from the contralateral side and sham-operated mice. Contrary to LAMP1, the amount of cathepsin-D detected in the cytosol of the *area penumbra* neurons increases compared with analogous contralateral regions and sham-operated mice (Figure 11A). However, when the count of cathepsin-D molecules was carried out selectively within lysosome vacuoles a massive decrease in cathepsin-D molecules was measured (Figure 11B). Remarkably, the magnitude of the decrease in vacuolar cathepsin-D surpasses the increase in cytosolic cathepsin-D as shown in graph of Figure 11C, where the ratio between cytosol and vacuole cathepsin-D is reported. This is consistent with a loss of lysosome compartmentalization of lysosome-related molecules (both LAMP1 and cathepsin-D) within *area penumbra* neurons. This is confirmed by the graph of Figure 11D where the amount of cathepsin-D-positive vacuoles is decreased similarly to vacuolar cathepsin-D molecules.

**Figure 12:**
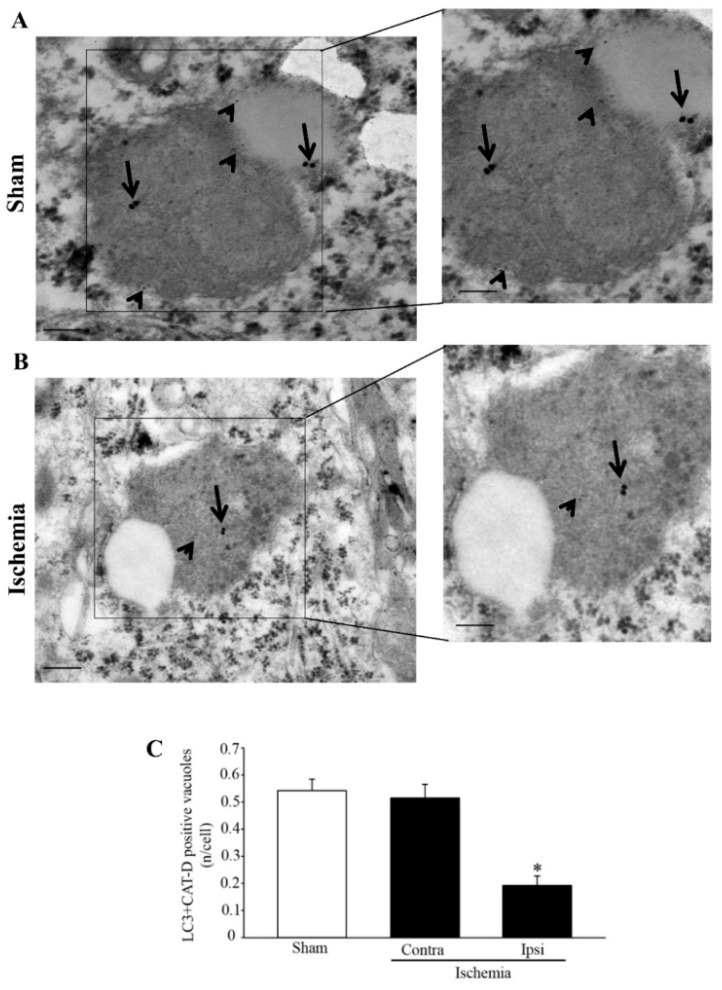
Microtubule-associated protein I/II-Light Chain 3 (LC3) + cathepsin-D (CAT-D) co-immuno-stained vacuoles decrease within the *area penumbra*. (**A**) shows representative TEM micrographs of LC3 + CAT-D co-immuno-stained vacuoles within a cortical neuron from a region homologous to the *area penumbra* from a sham-operated mouse (Sham), while (**B**) shows LC3 + CAT-D co-immuno-stained vacuoles within a cortical neuron from the *area penumbra* from an ischemic mouse (Ischemia). Arrows point to LC3 (20 nm) and arrowheads point to CAT-D (10 nm) immuno-gold particles within vacuoles. The inserts report LC3 + CAT-D immuno-gold particles within vacuoles at higher magnification. Graph (**C**) reports the number of LC3 + CAT-D immuno-gold double-stained vacuoles per cell from the *area penumbra* (Ipsi/Ischemia) compared with homologous regions from the contralateral side (Contra/Ischemia) and sham-operated mice (Sham). Values are given as the mean ± S.E.M. per cell counted in 120 cells in each group. * *p* ≤ 0.05 compared with other groups. CAT-D = cathepsin-D. Scale bar = 0.2 µm (**A**,**B**), 0.1 µm (inserts).

The effects of a loss of cathepsin-D-positive vacuoles and molecules area further expressed by co-staining vacuoles with LC3 and cathepsin-D within the *area penumbra* and analogous control regions as shown in representative pictures of Figure 12A,B, respectively. As reported in the graph of Figure 12C, a massive decrease in cathepsin-D- + LC3-positive vacuoles (interpreted as autophagolysisosomes as shown in Figure 1 and Figure 2) occurs within ischemic penumbra. This phenomenon surpasses the decrease in cathepsin-D-positive vacuoles (lysosomes), which was reported in the graph of Figure 11D. The decrease in cathepsin-D- + LC3-positive vacuoles (Figure 12C) mimics the decrease in LAMP1- + LC3-positive vacuoles (shown in Figure 10C). These data indicate that a failure of autophagolysosomes characterizes the *area penumbra*. Considering the decrease in both LAMP1- and cathepsin-D-positive vacuoles, this relies partly on a primary failure of the lysosome compartment. In fact, a molecular defect in lysosome-related particles and vacuoles within the *area penumbra* occurs. This adds on a primary defect in LC3-positive autophagosomes (Supplementary Figure S1B). These single events both contribute to occlude the occurrence of autophagolysosomes reported here (see Figure 2B for a scheme). 

### 2.7. Effects of MCAO on HSP70 and LAMP1 Immunofluorescence within the area penumbra

**Figure 13:**
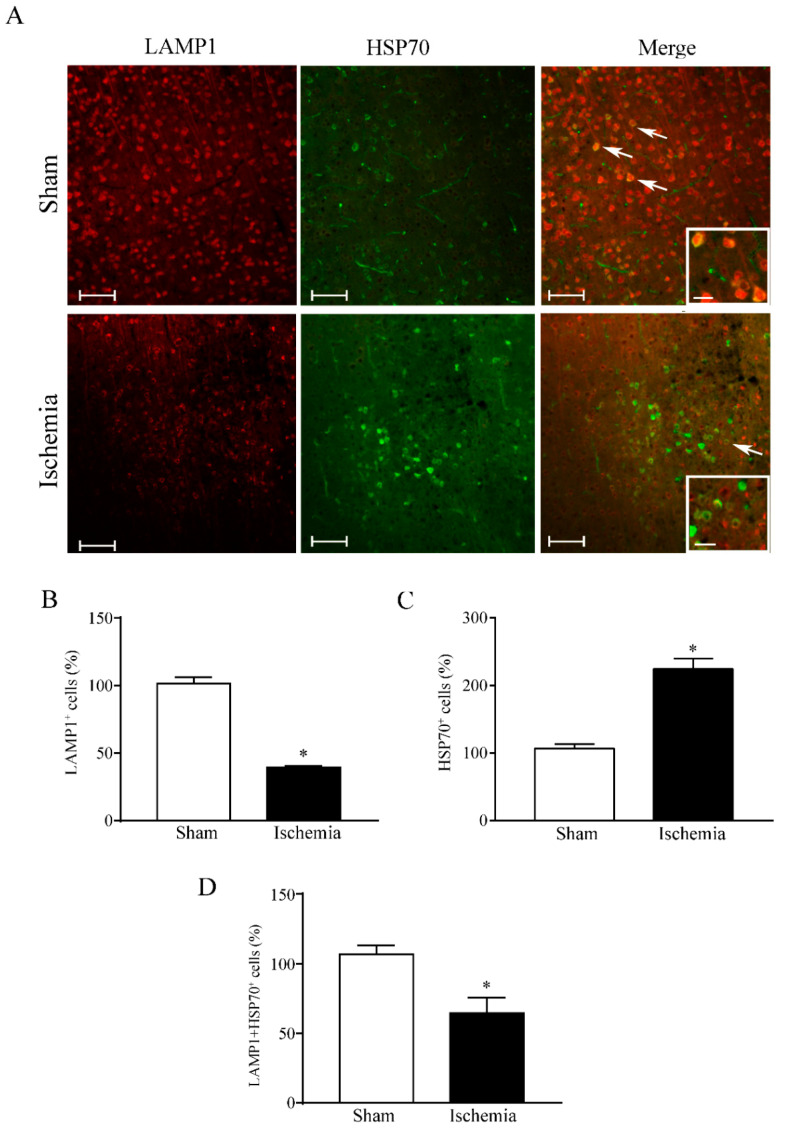
Within the *area penumbra*, lysosomal-associated membrane protein 1 (LAMP1) decreases, while Heat Shock Protein 70 (HSP70) increases and their co-localization is suppressed. (**A**) shows representative pictures show LAMP1 immunofluorescence (red), HSP70 immunofluorescence (green) and merge of both antigens. Arrows point to the merge of LAMP1 and HSP70. Graph (**B**) reports a decrease in LAMP1-positive cells within the ischemic *area penumbra* (Ischemia) compared with the control (Sham). Graph (**C**) reports the increase in HSP70-positive cells within the ischemic *area penumbra* (Ischemia) compared with the control (Sham). Graph (**D**) reports a massive decrease in LAMP1- + HSP70-positive cells within the ischemic *area penumbra* (Ischemia) compared with the control (Sham). Values are reported as percentage of control and values are given as the mean ± S.E.M. of 12 values for each group (n = 3 slices × N = 4 mice). * *p* ≤ 0.05 compared with other groups. Scale bar = 50 µm; 10 µm (inserts) (**A**).

Since the present manuscript provided partial evidence about a common fate of LC3 and HSP70, the study was extended to the lysosomal merging of HSP70. This is first documented by immunofluorescence, which confirms a decrease in LAMP1 within the *area penumbra*. The co-localization of LAMP1 with HSP70 within the *area penumbra* was suppressed compared with a sham mouse as shown in representative Figure 13A. This occurs even though HSP70 is markedly increased. In fact, while LAMP1 is markedly suppressed within the *area penumbra*, HSP70 increases (graph of Figure 13B,C, respectively). The loss of lysosomal merging of HSP70 is evidenced by the graph of Figure 13D where a massive decrease occurs for neurons co-stained for HSP70 and LAMP1. These findings substantiate a defect of LAMP1, which occurs within HSP70-immuno-positive neurons of the *area penumbra*.

### 2.8. Effects of MCAO on Concomitant HSP70 and LAMP1 Stoichiometry within the area penumbra

**Figure 14:**
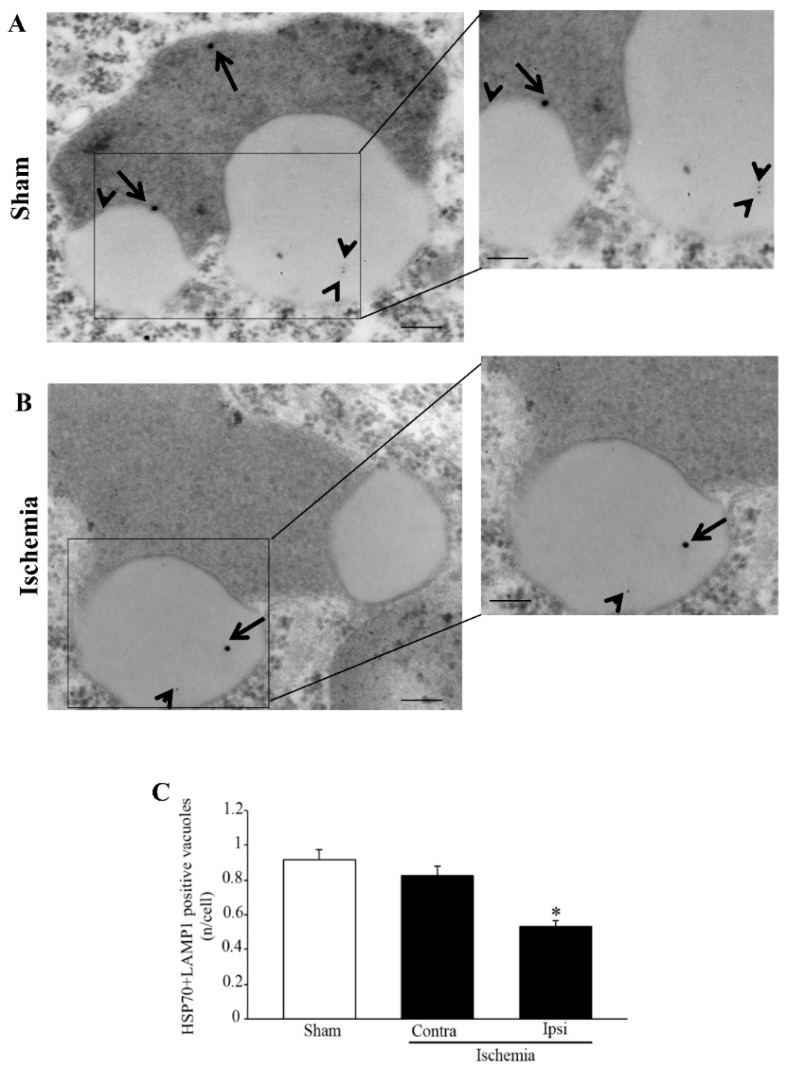
Heat Shock Protein 70 (HSP70) + lysosomal-associated membrane protein 1 (LAMP1) co-immuno-stained vacuoles decrease within the *area penumbra*. (**A**) shows representative transmission electron microscopy (TEM) micrographs of HSP70 + LAMP1 co-immuno-stained vacuoles within a cortical neuron from a region homologous to the *area penumbra* from a sham-operated mouse (Sham). (**B**) shows HSP70 + LAMP1 co-immuno-stained vacuoles within a cortical neuron from the *area penumbra* from an ischemic mouse (Ischemia). Arrows point to HSP70 (20 nm) and arrowheads point to LAMP1 (10 nm) immuno-gold particles within vacuoles. The insert report HSP70 + LAMP1 immuno-gold particles within vacuoles at higher magnification. Graph (**C**) reports the number of HSP70 + LAMP1 immuno-gold double-stained vacuoles per cell from the penumbra arewa (Ipsi/Ischemia) and homologous regions from the contralateral side (Contra/Ischemia) and from sham-operated mice (Sham). Values are given as the mean ± S.E.M. per cell counted in 120 cells from each group. * *p* ≤ 0.05 compared with other groups. Scale bar = 0.2 µm (**A**,**B**), 0.1 µm (inserts).

To confirm these data at the sub-cellular level, the specific cell compartments were assessed as shown in representative pictures of Figure 14 from both a sham and an ischemic mouse. Both HSP70 and LAMP1 are present within the same vacuole within a neuron from a sham-operated mouse (Figure 14A) and within a neuron from the *area penumbra* from an ischemic mouse (Figure 14B). The amount of co-localization is suppressed within the *area penumbra* compared with analogous regions in the contralateral hemispheres and from sham-operated mice (Figure 14C). These findings indicate that the co-localization of HSP70 and LAMP1 within the same vacuoles is significantly suppressed within the *area penumbra*.

### 2.9. Ultrastructural Stoichiometry from Dorsal area penumbras

All the data reported in the main body of the manuscript concerning the ultrastructural stoichiometry of each molecule are considering the ventral cortical penumbra region. All data were replicated within dorsal *area penumbras* for each molecule and these data are reported in Supplementary Figures. In detail, Supplementary Figures S2 and S3 reports LC3 and HSP70 within cytosol and vacuoles, which replicates data reported for the ventral area. Supplementary Figure S4 reports graphs counting the cytosol and vacuolar placement of LAMP1 and its co-localization with LC3, which replicates findings reported in the body of the text concerning the ventral area. Supplementary Figure S5 reports data concerning cathepsin-D immune-gold stoichiometry within cytosol and vacuoles and its co-localization with LC3. Finally, Supplementary Figure S6 reports data showing the co-localization of HSP70 and LAMP1 within vacuoles within the dorsal *area penumbra*. These data are consistent with findings reported in the main body of the text, which refer to the ventral penumbra area.

## 3. Discussion

The *area penumbra* corresponds to the slightly ischemic, peri-infarct area of the nervous matter, which expresses intensely the chaperone protein HSP70. In fact, this molecule is the classic marker of the region and it overlaps reliably with MRI-defined “*area penumbra*” [5]. Thus, HSP70 immuno-staining is routinely employed, in pre-clinical research to identify the *area penumbra*.

Recently, the staining of such an ischemic brain region has been implemented with other markers. Among these, proteins belonging to the autophagy machinery [8,9,10,16,17,18,19,20]. In fact, autophagy-related proteins increase within the *area penumbra* [8,9,10,14,21]. In the ischemic region, autophagy proteins appear to be consistently increased in various experimental models, despite the fact that their deleterious [11] or protective [12] effects are under debate [14]. Among these autophagy proteins, LC3 increases within the *area penumbra* as much as HSP70. Nonetheless, to our knowledge, no study so far has analyzed the co-expression of the classic marker HSP70 along with LC3 ex vivo or in vivo within the same brain area. 

In the present study, such an investigation was carried out by using a model of permanent occlusion of MCA [22]. Within this *area penumbra*, the autophagy protein LC3 was analyzed and quantified concomitantly with HSP70. In the first part of the study, evidence is provided that zonal, neuronal and subcellular placement of LC3 within the *area penumbra* mimics that measured for HSP70. This provides the first demonstration that LC3 increases in the very same zones of the ischemic cerebral cortex marked as the *area penumbra* by HSP70. The increase in these molecules is very similar, both concerning cortical zones and specific cells. In fact, the cells expressing HSP70 all merge with LC3-positive cells within the *area penumbra*. Co-expression of these molecules occurs in the very same neurons as confirmed by immuno-ultrastructural stoichiometry. Again, a remarkable overlapping occurs for LC3 and HSP70 concerning the subcellular compartments. In fact, in the control tissue, HSP70 preferentially localizes within LC3-positive autophagy vacuoles. Within the *area penumbra*, ischemia produces a shifting in the compartmentalization of both HSP70 and LC3 from vacuoles to cytosol. Within the same neurons, both HSP70 and LC3 molecules, while decreasing in the vacuoles, increase massively in the cytosol and the whole cell. The powerful vision provided by ultrastructural morphometry indicates that, within the *area penumbra*, LC3 (as well as HSP70) does not increase within neuronal vacuoles. It is rather the opposite, since the vacuolar placement of LC3 (as well as HSP70) is drastically reduced. Thus, the increase in LC3 (and HSP70) in the whole cell occurs within cytosol only, where LC3 may aggregate to produce hot fluorescent spots which had been interpreted as big stagnant autophagy vacuoles. The present study rules out an overexpression of LC3-immuno-positive vacuoles within *area penumbra* neurons. These findings strongly challenge the hypothesis, based on light microscopy, that within the *area penumbra* an excess of stagnant autophagosomes occurs, since here LC3-positive vacuoles are markedly reduced within the *area penumbra*.

The increase in LC3-positive hot spots is likely dependent on the clustering of LC3 in the cytosol, which may generate spots of immunofluorescence within ischemic penumbra contrasting with the mild fluorescence of controls. The hypothesis that LC3 fluorescent spots in *area penumbra* neurons may not correspond to autophagy vacuoles was already formulated [8,10,15]. In the present manuscript, we provide actual evidence witnessing a decrease in LC3-positive autophagosomes and a decrease in the amount of LC3 molecules within each autophagosome. Based on these findings, it is unlikely that enhanced autophagy may occur in the *area penumbra* compared with the control tissue. In fact, LC3 increases in the cell but it is massively reduced in the vacuoles, where it is needed to induce autophagy clearance. Similarly, the present findings lead to the re-interpretation of previous data based on TEM observations of giant autophagy-like vacuoles [23,24,25]. In fact, big vacuoles occur but without LC3 particles. Since LC3-positive autophagy vacuoles are rather decreased within the *area penumbra*, the spots of LC3 immunofluorescence may be due to cytosolic non-vacuolar LC3 protein aggregates [26,27]. In fact, in the present study, a considerable increase in cytosolic LC3 is confirmed. 

A major finding of the present study concerns the merging of LC3 and HSP70 immuno-staining concerning zonal, cellular and sub-cellular compartments. In fact, the very same cortical zones and neurons increase the staining for both antigens. Since the expression of HSP70 within the *area penumbra* is considered to be protective [28,29], this suggests analogous protective effects of LC3. In fact, despite contradictory findings, a number of studies suggest that promoting LC3 expression and compartmentalization protects neurons of the *area penumbra* [13]. Similarly, it would be important to analyze whether increased protection induced by the overexpression of HSP70 relies on its placement within autophagosomes, rather than whole cell amount. This way, considering that HSP70 is compartmentalized within LC3-positive autophagy vacuoles may lead to the reconsideration of the role of this chaperone protein as key component of the autophagy machinery. In fact, HSP70 exerts powerful protection against oxidative species generated during ischemia. The present data indicate that combining both antigens may provide a more sensitive marker to detect *area penumbra* cells. The sole morphological evidence of a remarkable merging between HSP70 and LC3 within the *area penumbra* neurons needs to be implemented by functional data to explore whether a synergism takes place. 

A key point to interpret the autophagy status in the *area penumbra* consists of establishing the fate/progression of LC3-positive autophagy vacuoles. Similarly, due to the co-localization of HSP70, an additional question concerns the fate of HSP70-positive vacuoles. The merging of autophagosomes with lysosomes is key to understand the cell clearance. Zhang et al. (2021) [15] postulated that increased expression of autophagy markers (believed to be generated by increased autophagosomes) may engulf the lysosomal compartment, thereby producing a paradox defect in autophagolysosomal degradation [15]. The present study instead indicates a combined failure of both autophagosome and lysosome compartments. In fact, autophagy vacuoles are diminished and their content in LC3 is decreased. Similarly, the lysosome compartment is depleted of LAMP1. Even cathepsin-D, which is increased in the cell, it is suppressed in the vacuoles. The present finding may be in line with further observation of Zhang et al. (2021) [15], who indeed noticed that, during ischemia, a lysosomal dysfunction is evident by a specific lysosomal alteration.

These findings indicate the lysosomal compartment as a kernel to solve the issue. 

In fact, in the second part of the present study, evidence is provided which shows that both LC3 and HSP70 within *area penumbra* neurons fail to merge with cathepsin-D- or LAMP1-positive vacuoles, which is consistent with the occurrence of a failure in the occurrence of autophagolysosomes. When specific stoichiometry for LAMP1 and cathepsin-D is carried out, a primary defect of both proteins is detected within vacuoles. These findings lead to the hypothesis that within the peri-infarct region a failure of the whole autophagolysosome machinery takes place. This depends on multiple phenomena which are reported in the scheme of Figure 2A. At first, a defect of LC3 molecules within autophagosome occurs along with the dissipation of LC3 in the cytosol. This is accompanied by a decrease in LAMP1 and cathepsin-D within the vacuoles. The decrease in the merging between autophagy and lysosome proteins exceeds the defect of the specific proteins and vacuoles. This suggests an additional defect, which may be hypothesized consisting of a defective merging of autophagosomes with lysosomes, as postulated in the scheme of Figure 2A. 

Remarkably, in the present study, evidence emerges which entraps HSP70 in such a dysfunctional cell clearance within *area penumbra* neurons. In fact, in the *area penumbra*, the merging of vacuolar HSP70 within LAMP1-positive vacuoles is defective. The loss of such a synergism is likely to induce deleterious effects. In fact, the severe depletion of both lysosome and autophagosome is replicated by a loss of their chaperone (HSP70) component. This loss of compartmentalization may be in need to be rescued to improve neuronal survival following ischemia.

The present data provide direct evidence for a defective compartmentalization of chaperone, autophagy and lysosomal molecules, which could not be detected by using light microscopy alone. In fact, a confounding point, when assessing autophagosomes and lysosomes by light microscopy, is the potential bias of interpreting puncta as a witness of vacuoles. This applies to LC3 spots of immunofluorescence (as discussed above) as well as to the measurement of lysosomal proteins. In fact, this is also the case of the lysosome-associated membrane protein LAMP1 or lysosomal enzymes such as cathepsins. All these proteins may not necessarily occur site-specifically within autophagosome or lysosome compartments. 

In fact, we measured that here, in ischemic conditions, the amount of both LC3 compartmentalized within autophagosome and cathepsin-D compartmentalized within lysosomes diminishes, while these proteins dramatically increase in the cytosol. In the case of cathepsin-D, this may lead to non-controlled digestion of cell substrates, which may produce autophagy-dependent cell death. Indeed, it is a loss of enzyme compartmentalization which generates the damage. The issue of preserved autophagosomes and lysosomes include the facet of compartmentalization of specific molecules. The loss of compartmentalization appears a key point. In fact, during ischemia a dissociation may occurs, where the increase in specific proteins does not cope with their placement within appropriate cell compartments. Instead, compartmentalization of molecular pathways is essential in providing appropriate activity. In ischemic cells, the LC3 and cathepsin-D level are increased, but their compartmentalization is suppressed (Schemes of Figure 1 and Figure 2A,B). This is also the case of LAMP1, which loses its vacuolar placement. In the present study, evidence is provided which shows that HSP70, which in the control neurons mimics the compartmentalization of LC3, is dissipated from autophagy vacuoles. Within the *area penumbra*, HSP70 is no longer able to merge with lysosome proteins. The loss of interaction between these proteins, though selective protein and organelle degradation, is a massive impairment, which is required for cell clearing machinery to be effective. In line with this, specific studies are needed to address whether compounds, such as rapamycin which promotes LC3 vacuolar placement and protects against ischemia [30], may also shift HSP70 and cathepsin-D within vacuoles and promote the merging of autophagosome and lysosome compartments.

## 4. Materials and Methods

### 4.1. Permanent Focal Ischemia in Mice 

Adult C57BL/6 male mice (Charles River, Middlesex County, MA, USA) weighing 25 g were housed under controlled conditions (ambient temperature, 22 °C; humidity, 40%) on a 12 h light–dark cycle with food and water ad libitum. The experimental protocol was approved by the Ethical Committee of Neuromed Institute (Pozzilli, Italy) and further supervised by The Italian Ministry of Health (Authorization number 1194/2020-PR). Mice were anaesthetized with chloral hydrate (400 mg/kg, i.p.). An incision was made between the outer canthus of the eye and the external auditory meatus and the temporal muscle was bisected and retracted to expose the external temporal aspect of the skull to produce a small hole (0.5 mm^2^) via drill-operated craniotomy; thus, MCA was identified and exposed using stereomicroscopy [7,31]. The deepest bone layer was preserved to avoid drill penetration through dura mater leading to the risk of cortical mechanical and/or thermal damage. In fact, such a layer is removed subsequently, through a careful manual procedure to reach out the distal MCA, which was occluded by electro-coagulation. After this procedure, the temporal muscle and overlying skin bands were sutured. Body temperature was monitored during surgery through a rectal probe, which was connected in feed-back to the surgery pad. This allows us to keep the body temperature steady at 37 °C. Sham-operated mice undergo the same procedure, but MCA cauterization. In fact, the terms “sham” or “sham-operated”, refer to those mice which undergo the surgical procedure aimed at producing ischemia. In these mice, all surgical steps were carried out but the occlusion of the middle cerebral artery (MCAO). This indicates that surgery was carried out without producing the occlusion of the middle cerebral artery. The group of sham mice is useful to compare the effects which purely depend on the ischemia (“ischemic mice”) and are not related with other steps in the surgical procedure (“sham” or “sham-operated mice”). Thus, comparisons between MCAO and sham-operated mice allows us to interpret all changes observed in the MCAO group as specifically induced by the occlusion of the middle cerebral artery. 

All mice are sacrificed at 24 h following MCAO and brains are dissected for analysis.

### 4.2. Light Microscopy

Brains were immediately placed into a Carnoy’s fixing solution composed of ethyl alcohol (60%), acetic acid (10%) and chloroform (30%) for histochemistry and immunofluorescence. Twenty-four hours later, brains are placed in 70% ethanol to be wax-embedded. The brains were cut into 10 μm-thick coronal slices at microtome (Leica Microsystem, RM2125, Milan, Italy). Each slice was regularly spaced 550 µm. Slices were used for histochemical and immunofluorescence analysis.

For histochemical analysis, slices were de-waxed and processed for staining with thionin (a kind of Nissl staining) to frankly assess infarct and peri-infarct regions. 

As shown in representative Figure 3, the cortical peri-infarct area is evident following thionin staining as a region, where a sudden chromatic switch from the pale white frankly necrotic area to the blue normal Nissl-stained brain matter takes place. The cortical infarct area is well characterized as the pale region delimited by a dorsal and ventral peri-infarct area. Since the study focuses on the peri-infarct region as the presumed site of *area penumbra*, the cortex being analyzed for morphology is restricted to the area delimited by the red squares of Figure 3. Analogous regions were selected from the contralateral hemisphere and from corresponding cortical regions from sham-operated mice. Within these borderline regions, whole slices were cut for immunofluorescence; small fragments within these red squares were further dissected for electron microscopy and ultrastructural stoichiometry.

### 4.3. Immunofluorescence

Paraffin-embedded mouse brain sections (10 μm) were used for immunofluorescence, which was quantified by measuring the number of immunofluorescent cells from three slices per mouse (spaced 550 µm apart, N = 4 for each experimental group). Slices were obtained from the peri-infarct cortical regions to encompass the *area penumbra*. Three serial coronal slices were selected from the ventral border at the middle of the rostro-caudal extent of the ischemic region (ranging from bregma +1.94 mm back to bregma +0.14 mm). These specimens were used for staining with rabbit polyclonal anti-LC3B (Santa Cruz Biotechnology, Dallas, TX, USA), monoclonal mouse anti-LAMP1 (Genetex, Irvine, CA, USA) and monoclonal rabbit anti-HSP70 or monoclonal mouse anti-HSP70 (Thermo Fisher Scientific, Waltham, MA, USA and Abcam, Cambridge, UK, respectively). Slices were treated with normal sera for 1 h (10% in TBS). Then, they were incubated overnight (4 °C) with primary antibody anti-LAMP1, anti-HSP70 and anti-LC3B (for details see Table 1) and then for 1 h with secondary AlexaFluor 488/CY3-coupled anti-mouse or anti-rabbit IgG (for detail see Table 1). Control staining was performed without primary antibodies.

Immunofluorescence was assessed considering the ratio between positive cells and the background fluorescence measured in the corpus callosum by using a Zeiss AxioPhot2 microscope equipped with a digital video camera. Images were acquired at low magnification (10×). Results are given as percentage of the mean ± S.E.M. of 12 values for each group (n = 3 slices × N = 4 mice). Unpaired, two-tailed T-test was used for statistical comparison of collected data. Hypothesis H_0_ (μ1 = μ2) was rejected when *p* ≤ 0.05.

### 4.4. Transmission Electron Microscopy

Blocks from the cerebral cortex (1 mm × 1.3 mm × 1 mm) were dissected from the ipsilateral and contralateral cortex from ischemic mice (N = 6) and from both hemispheres from sham-operated mice (N = 6). From each side of each mouse, both the ventral border (Figures in the body of the text) and the dorsal border (Supplementary Figures) of the peri-infarct area were analyzed. Therefore, the number of areas in the site of the *area penumbra* corresponds to 2 (dorsal and ventral) ipsilateral for each mouse (N = 6) undergoing ischemia for a total of 6 × 2 = 12 *area penumbra*. Similarly, 12 corresponding regions were analyzed from the contralateral side of the same mice, which brings the number of regions from ischemic mice to 24. In addition, since 6 sham-operated mice were used, each one carrying a bilateral dorsal and ventral region, a total of 24 regions were also examined in sham-operated mice. This corresponds to 48 regions owing the very same placement, which were counted from ischemic and sham-operated mice. Corresponding regions from the two hemispheres of sham-operated mice were overlapping concerning all measurements (pilot experiments). This allowed us to pool left and right specimens from sham-operated mice in a total of 6 ventral and 6 dorsal areas to homogenize the number of specimens from each group.

In fact, the use of sham-operated mice in addition to the contralateral (non-ischemic side) works as an additional control to rule out systemic and widespread compensatory changes altering protein expression (LC3, HSP70, cathepsin-D and LAMP1) all over the brain following focal ischemia. In fact, this may indirectly influence the contralateral hemisphere through systemic effects or widespread subcortical compensatory innervation. When such a phenomenon occurs, even slightly, on the contralateral side, this may reduce the analytical power of measuring ischemia-induced antigen expression compared with the control tissue.

For TEM analysis, tissue samples were fixed in a solution containing paraformaldehyde 2.0%, and glutaraldehyde 0.1%, in 0.1 M PBS, pH 7.4. Then, specimens were dissected and immersed in the same solution overnight at 4 °C. After washing in PBS (0.1 M), samples were post-fixed in 1% osmium tetroxide (OsO_4_) for 1 h at 4 °C.

Tissue blocks were dehydrated in a serial gradient of ethanol solutions (30%, 50%, 70%, 90% and 95% for 5 min, and 100% for 60 min) to be embedded in epoxy resin. The concentration of the fixing and post-fixing solutions along with the use of the epoxy embedding resin were validated in previous studies using immuno-gold at ultrastructural morphometry [32,33]. In fact, the combination of aldehydes, OsO_4_, and epoxy resin allows a minimal epitope covering while preserving sub-cellular architecture [34,35,36]. This way, small sub-cellular structures such as vacuoles were still evident, steady and electron dense (Figure 2).

Ultra-thin slices were used both for plain and post-embedding immuno-gold electron microscopy. Ultra-thin slices were stained with uranyl acetate and lead citrate to be visualized at a Jeol JEM SX100 electron-microscope (Jeol, Tokyo, Japan). Before cutting ultra-thin sections at ultra-microtome, semi-thin slices were carried out from blocks embedded in epoxy resin by using the same ultra-microtome, which was set at a width of 700 nm per slice. This was carried out in order to orient the ultra-structural scanning of the *area penumbra* and allowing the identification of specific cell types to be analyzed at TEM.

### 4.5. Post-Embedding Immuno-Gold Microscopy

Ultra-thin sections, width = 90 nm, collected on nickel grids were processed for proteins detection by using primary antibodies detailed in Table 1. This experimental setting allows us to detect detailed morphometry of organelles, where membranes appear as sharply contrasting, while detecting immuno-gold particles to mark specific proteins. This way, the protein amount within specific cell compartments, such as vacuoles, cytosol and mitochondria, could be counted. When the distribution of LC3 and HSP70 was measured within the same vacuoles, the distance between LC3 and HSP70 molecules was calculated in co-stained vacuoles. The various distances of co-localization were considered and a normal distribution was assessed for 95% of distances. In this context, no specific grouping of different molecules at close or remote distance could be detected. In contrast, the LC3 molecule is placed according to a group pattern, which is compatible with LC3 aggregates.

Ultra-thin slices were layered on droplets of aqueous sodium metaperiodate (NaIO_4_) for 30 min at 22 °C to remove OsO_4_ for protein unmasking, while keeping optimal preservation of cell ultrastructure [35]. After rinsing in PBS, grids were exposed to drops of blocking solution (10% goat serum and 0.2% saponin in PBS) for 20 min at 22 °C. Grids were incubated in a humidified chamber overnight at 4 °C with either single or combined primary antibodies (rabbit polyclonal anti-LC3, Abcam; mouse monoclonal anti-HSP70, Abcam; mouse monoclonal anti-cathepsin-D, Sigma Aldrich, Milan, Italy; or mouse monoclonal anti-LAMP1, Abcam) in ice-cold PBS solution containing 1% goat serum and 0.2% saponin. After washing in cold PBS, ultra-thin slices were incubated with gold-conjugated secondary antibodies (both 10 nm and 20 nm immuno-gold particles, when double-staining was carried out). Antibodies were diluted 1:20 within a blocking buffer (containing 1% goat serum and 0.2% saponin in PBS) for 1 h at 22 °C. Counts of immuno-gold particles (10 nm and 20 nm) were carried out by using a low magnification (8000×), still allowing immuno-gold particles to be counted and whole cell organelles to be identified [37,38]. To count immuno-gold particles in cortical cells, measurements started from the corner of a grid square to proceed by scanning the whole grid square [39]. In the present study, the number of immuno-gold particles was counted for each primary antibody within the cytoplasm and within the vacuoles for each neuron (as visualized in Figure 2).

A total of 20 cortical neurons were counted from each region from each mouse (N = 6), leading to 120 cells for each region per group.

### 4.6. Statistical Analysis

Immunofluorescent cells (number/mm^2^) were counted from brain slices stained with LC3 and/or HSP70, LC3 and/or LAMP1 and LAMP1 and/or HPS70. Slices spaced 550 µm apart were calculated for each region (*area penumbra* or homologous regions from sham-operated mice). Three slices were counted for each animal. Values for neuronal density (number of neurons/mm^2^) were calculated within an area of 82,500 μm^2^. Results are given as percentage of positive cells compared with the control (sham-operated mice). Results are given as percentage of the mean ± S.E.M per group. Inferential statistics to compare groups was carried out by using a Student’s *t*-test (H_0_, null hypothesis, was rejected when *p* ≤ 0.05).

For electron microscopy, the assessment of vacuoles and measurement of immuno-gold particles within vacuoles and cytosol was carried out according to Lenzi et al. (2016) [34]. For ultrastructural stoichiometry, the following items were counted: (i) number of unstained vacuoles within cytosol; (ii) number of LC3, HSP70, cathepsin-D and LAMP1, immuno-gold particles within cytosol; (iii) number of LC3, HSP70, cathepsin-D and LAMP1 immuno-gold particles within each vacuole per cell; and (iv) number of vacuoles where co-localization occurs for: (i) LC3 and HSP70, (ii) LC3 and cathepsin-D, (iii) LC3 and LAMP1 and (iv) HSP70 and LAMP1. Ultrastructural morphometry was also used to calculate the ratio between the measurements reported above. This was expressed as follows: (i) the ratio between LC3 immuno-gold particles within vacuoles and cytosolic LC3 immuno-gold particles; (ii) the ratio between HSP70 immuno-gold particles within vacuoles and HSP70 immuno-gold particles within cytosol; (iii) the ratio between cathepsin-D immuno-gold particles within vacuoles and cathepsin-D immuno-gold particles within cytosol; and (iv) the ratio between LAMP1 immuno-gold particles within vacuoles and LAMP1 immuno-gold particles within cytosol.

Each count was carried out by two blind observers. Data are given as the mean ± S.E.M.

Data were compared by using one-way ANOVA with Fisher’s Test. Null Hypothesis (H_0_) was rejected when *p* ≤ 0.05.

## 5. Conclusions

The present manuscript reports the amount and placement of specific molecules which characterize *area penumbra* neurons and mark the autophagosome and lysosome compartments (Figure 1).

Within the *area penumbra*, protein amount and compartmentalization varies according to the arrows reported in the scheme of Figure 2A (control) and Figure 2B (ischemia). In detail, within *area penumbra* neurons, the autophagy-related molecule LC3 increases. This overall increase is specifically due to the cytosolic amount of LC3, while the amount placed within vacuoles is decreased (Figure 2B and Figure 6). Similar findings concern the molecule HSP70, which increases in *area penumbra* cells due to a massive increase in the cytosol, while its sub-cellular placement within vacuoles is reduced (Figure 2B). These combined effects produce a massive decrease in co-localization of LC3 + HSP70 within the autophagosome (Figure 2B and Figure 6).

A similar decrease in vacuolar compartmentalization occurs for the lysosome molecule LAMP1, which is decreased both in lysosome vacuoles and cytosol of *area penumbra* neurons (Figure 2B and Figure 9). The lysosome-specific enzyme cathepsin-D increases in the whole cell of the *area penumbra* due to a massive increase in the cytosol, while its specific lysosome compartmentalization is reduced (Figure 2B and Figure 11).

This indicates that ischemia produces a dissipation of specific molecules from their own specific physiological compartments. 

Altogether, these findings generate the concept that in ischemia, lysosomal function and autophagosome formation are impaired [17].

## Figures and Tables

**Table 1:** Primary and secondary antibodies used in this study.

Antibody	Distributor	Catalog Number	RRID	Concentration
Rabbit polyclonal anti LC3B	Santa Cruz Biotechnology, Dallas, TX, USA.	Cod. SC28266	AB_2137719	1:50
Rabbit polyclonal anti LC3B	Abcam, Cambridge, UK.	Cod. 128025	AB_11143008	1:50
Monoclonal rabbit anti HSP70	Thermo Fisher Scientific, Waltham, MA, USA.	Cod. 33-3800	AB_2533116	1:100
Monoclonal mouse anti HSP70	Abcam, Cambridge, UK.	Cod. Ab5439	AB_304888	1:20
Rabbit anti-Cathepsin-D	Novus Biological, Milan, Italy	Cod. NBP1-85562	AB_11014649	1:1000
Monoclonal mouse anti Cathepsin-D	Sigma Aldrich, Milan, Italy	Cod. C0715	AB_258707	1:10
Monoclonal rabbit anti-LAMP1	Sigma Aldrich, Milan, Italy	Cod. Ab-91170	AB_2665831	1:1000
Monoclonal mouse anti-LAMP1	Genetex, Irvine, CA, USA.	Cod. GTX634336	AB_2888439	1:100
Monoclonal mouse anti-LAMP1	Abcam, Cambridge, UK.	Cod. AB25630	AB_470708	1:50
Alexafluor 488 anti-mouse	Thermo Fisher Scientific, Waltham, MA, USA.	Cod. A21202	AB_141607	1:100
Donkey × Rabbit CY3	Millipore, Burlington, MA, USA.	Cod. AP182C	AB_92588	1:300
Goat Anti-Rabbit IgG Antibody, 20 nm Gold Conjugated	Bbi solutions, Edinburgh, UK	Cod. EM GAR20/0.25	AB_1769136	1:50
Goat Anti-Mouse IgG Antibody, 10 nm Gold Conjugated	Bbi solutions, Edinburgh, UK	Cod. EM GAR10/0.25	AB_1769128	1:50
Fluorescein anti-Rabbit	Vector Labs, D.B.A., Milano, Italy	FI-1000-1.5	AB_2336197	1:100
Rabbit polyclonal anti HSP70	Cell signaling, Danvers, MA, USA.	4872S	AB_2279841	1:1000
Mouse anti beta actin	Sigma Aldrich, Milan, Italy	A1978	AB_476692	1:25,000
Goat Anti Rabbit	Millipore, Burlington, MA, USA.	401-393	AB_437797	1:3000
Goat Anti Mouse	Millipore, Burlington, MA, USA.	401-215	AB_10682749	1:3000

## Data Availability

The data that support the findings of this study are available from the corresponding author upon reasonable request.

## Supplementary Materials

The following supporting information can be downloaded at: https://www.mdpi.com/article/10.3390/molecules27103122/s1, Figure S1: Ultrastructural morphometry of HSP70- and LC3-positive vacuoles; Figure S2: Ultrastructural morphometry of vacuoles, LC3 and HSP70 within the dorsal *area penumbra*; Figure S3: Within the dorsal *area penumbra*, HSP70 and LC3 shift from vacuole to cytosol; Figure S4: Within dorsal *area penumbra* cells, LAMP1 molecules are reduced and dispersed from vacuoles; Figure S5: Within dorsal *area penumbra* cells, cathepsin-D (CAT-D) molecules are reduced and dispersed from vacuoles; Figure S6: HSP70 + LAMP1 co-immuno-stained vacuoles decrease within the dorsal *area penumbra*.
