# Supplementary material for: Within the Ischemic Penumbra, Sub-Cellular Compartmentalization of Heat Shock Protein 70 Overlaps with Autophagy Proteins and Fails to Merge with Lysosomes
Source: Molecules. 2022 May 13;27(10):3122. doi: 10.3390/molecules27103122 (PMC9144499; doi:10.3390/molecules27103122)
Supplement: Supplementary file 1 [file molecules-27-03122-s001.zip › molecules-1705434-supplementary.pdf]

Supplementary Figures

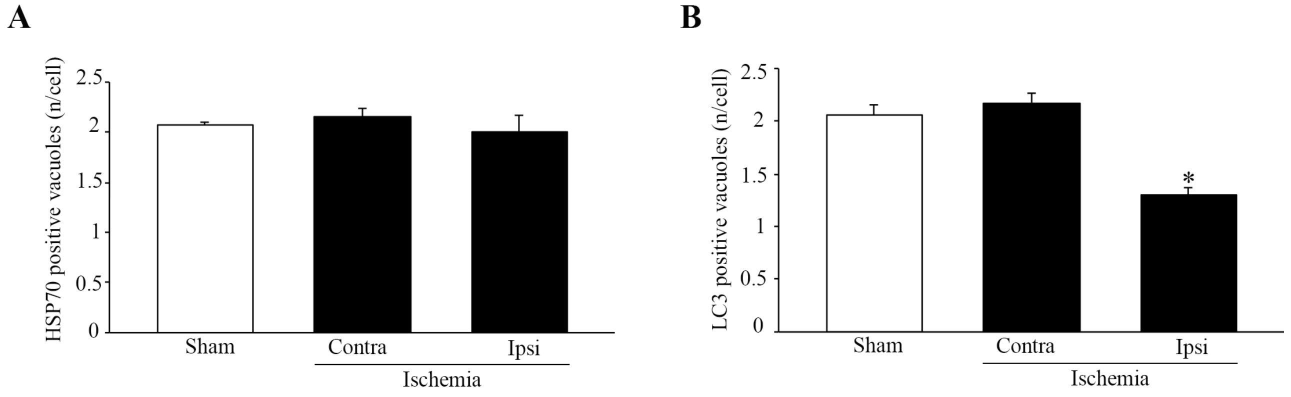

Figure S1. Ultrastructural morphometry of Heat Shock protein 70 (HSP70) and microtubule-associated protein I/II-Light Chain 3 (LC3) positive vacuoles.

The number of HSP70 positive vacuoles per cell remains steady in the ventral area penumbra (Ipsi/Ischemia) compared with the number counted in the homologous regions from the contralateral side (Contra/Ischemia) and from sham-operated mice (Sham) as reported in graph **A**. Graph **B** reports a decrease in the number of LC3 positive vacuoles per cell in area penumbra (Ipsi/Ischemia) compared with the number counted in the homologous regions from the contralateral side (Contra/Ischemia) and from sham-operated mice (Sham). Values are given as the mean±S.E.M. per cell from a total of 120 cells for each group. *P≤0.05 compared with other groups.

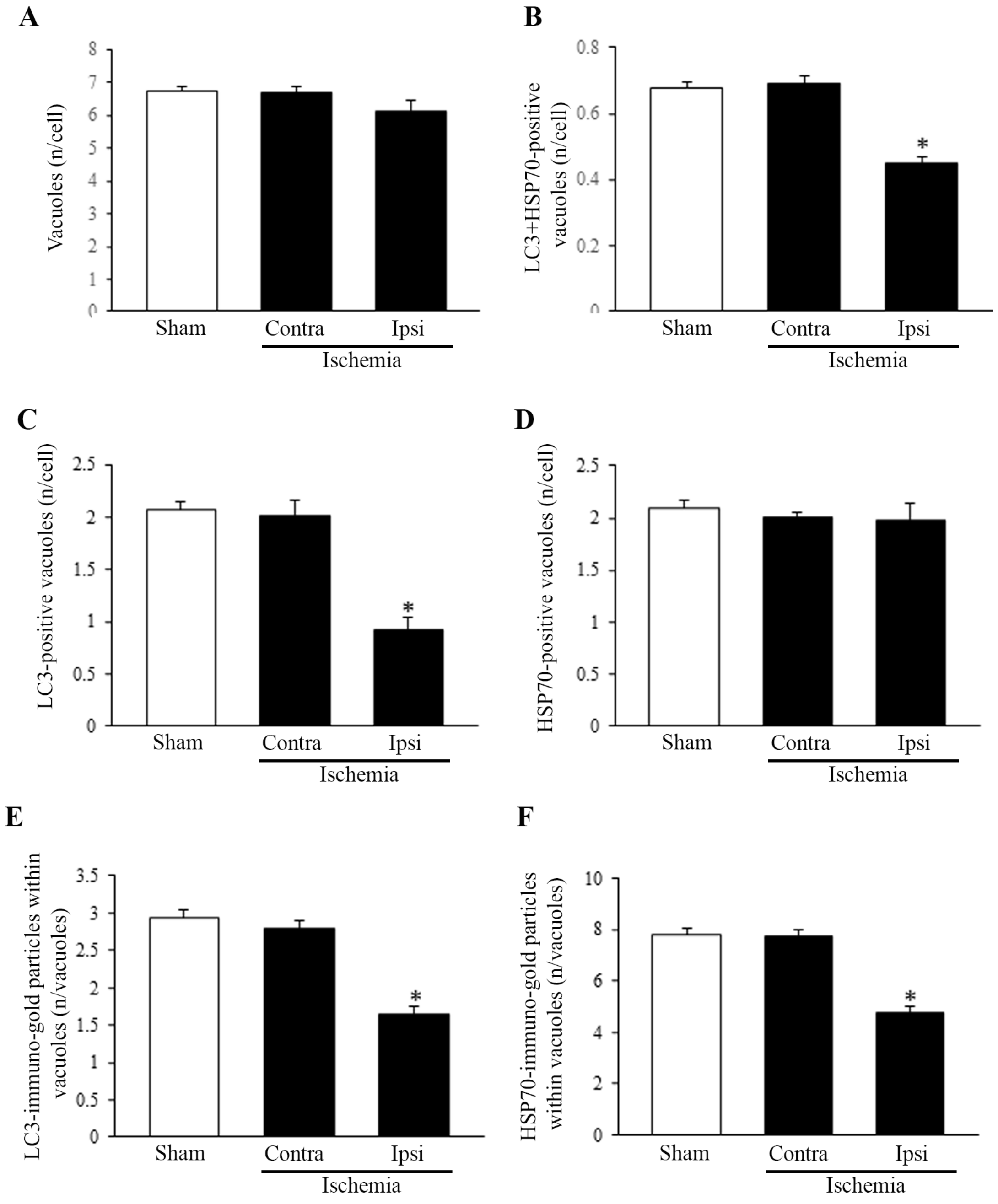

Figure S2. Ultrastructural morphometry of vacuoles, microtubule-associated protein I/II-Light Chain 3 (LC3) and Heat Shock protein 70 (HSP70) within dorsal area penumbra.

Graph (**A**) reports the number of vacuoles per cell. Graph (**B**) reports the number per cell of LC3+HSP70 positive vacuoles. Graphs (**C**) and (**D**) report the number of LC3 or HSP70 positive vacuoles respectively. Graphs (**E**) and (**F**) report the amount of LC3 or HSP70 particles within vacuoles, respectively. All values are counted in sham-operated mice (Sham), in the side contralateral to ischemia (Contra/Ischemia) and within area penumbra (Ipsi/Ischemia). Values are reported as the mean±S.E.M. of immuno-gold particle counted in each cell from a total of 120 cells for each group. *P≤0.05 compared with other groups.

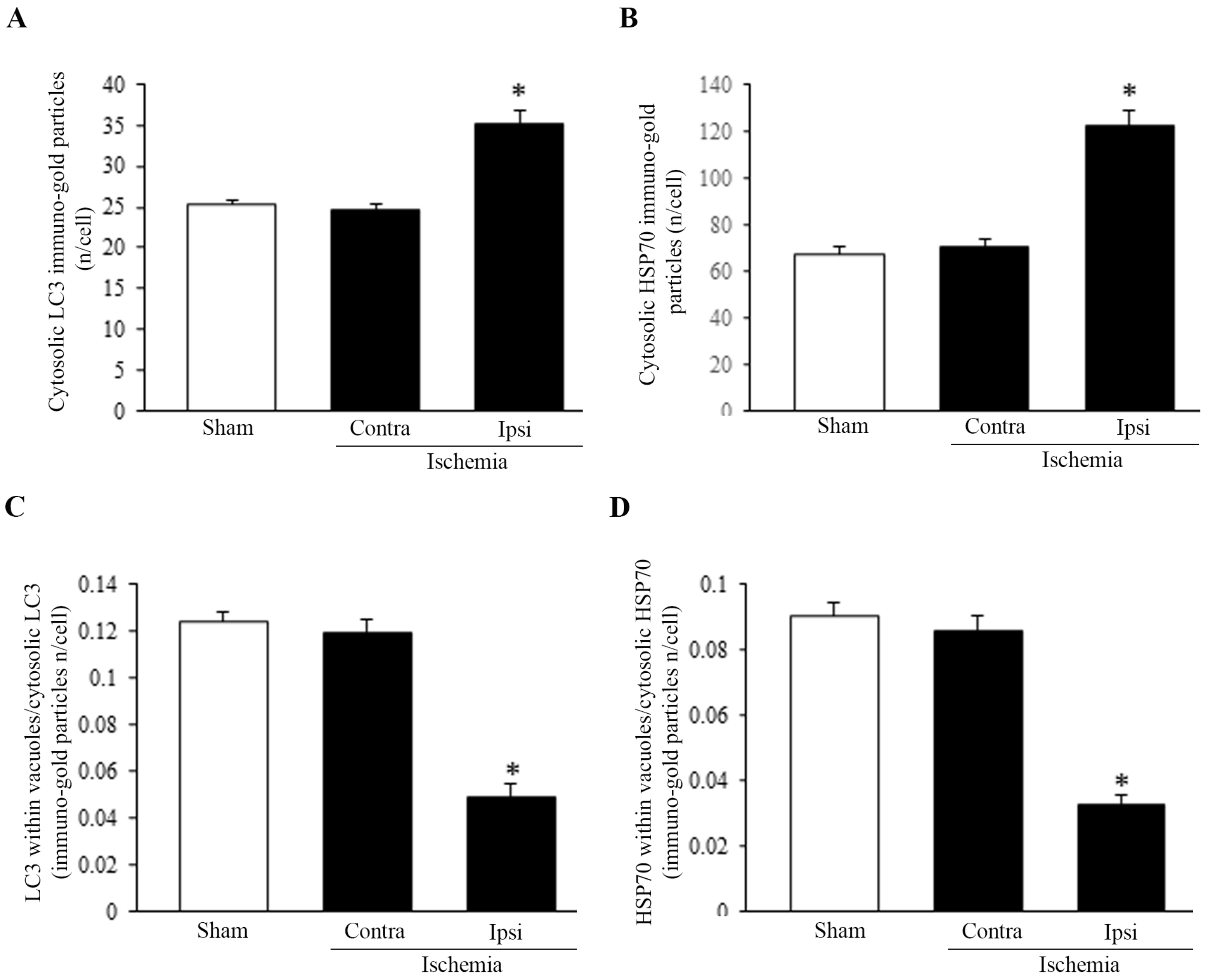

Figure S3. Within dorsal area penumbra Heat Shock protein 70 (HSP70) and microtubule-associated protein I/II-Light Chain 3 (LC3) shift from vacuole to cytosol.

Graphs on the left side report the amount of LC3 particles within cytosol (**A**), and the ratio between vacuolar and cytosolic LC3 particles (**C**). Similarly, graph on the right side report the amount of HSP70 particles within cytosol (**B**), and the ratio between vacuolar and cytosolic HSP70 particles (**D**). Particles are counted in sham-operated mice (Sham), in the side contralateral to ischemia (Contra/Ischemia) and within area penumbra (Ipsi/Ischemia). Values are reported as the mean±S.E.M. of immuno-gold particle counted in each cell from a total of 120 cells for each group. *P≤0.05 compared with other groups.

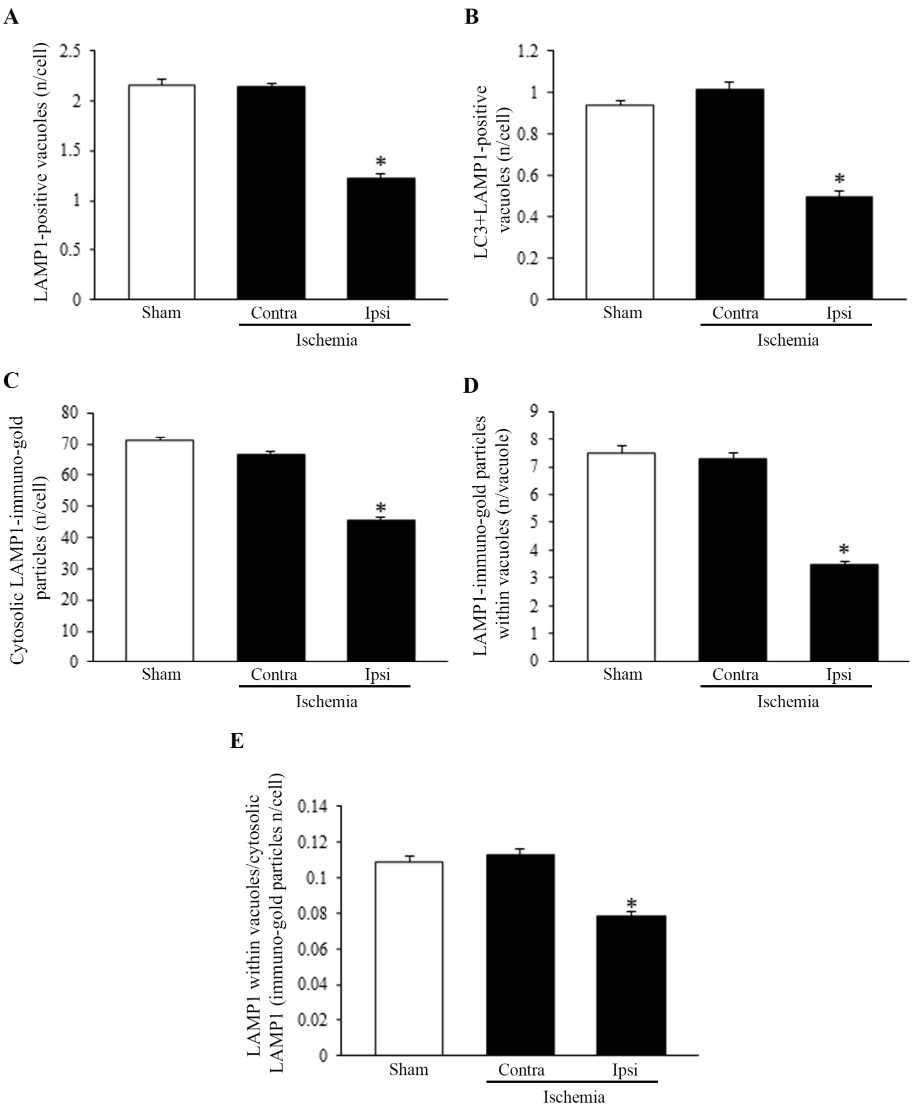

Figure S4. Within dorsal area penumbra cells Lysosomal associated Membrane Protein 1 (LAMP1) molecules are reduced and dispersed from vacuoles concomitantly to a reduction in microtubule-associated protein I/II-Light Chain 3 (LC3)+LAMP1 co-immuno-stained vacuoles.

Graph **A** reports the decrease in the number of LAMP1 positive vacuoles per cell. Graph **B** reports the decreased of LC3+LAMP1 positive vacuoles per cell. Graph **C** reports a reduced amount of LAMP1 molecules counted in the cytosol per cells. Graph **D** reports a massive decrease in the amount LAMP1 molecules counted within vacuoles per cell. Graph **E** reports a decrease in the ratio between vacuolar and cytosolic amount of LAMP1 molecules per cell. All graphs report the ultrastructural morphometry for LAMP1 and LC3+LAMP1 positive vacuoles from dorsal area penumbra (Ipsi/Ischemia) and from the side contralateral to ischemia (Contra/Ischemia) and from sham-operated mice (Sham).Values are reported as the mean±S.E.M. per cell from a total of 120 cells for each group. *P≤0.05 compared with other groups.

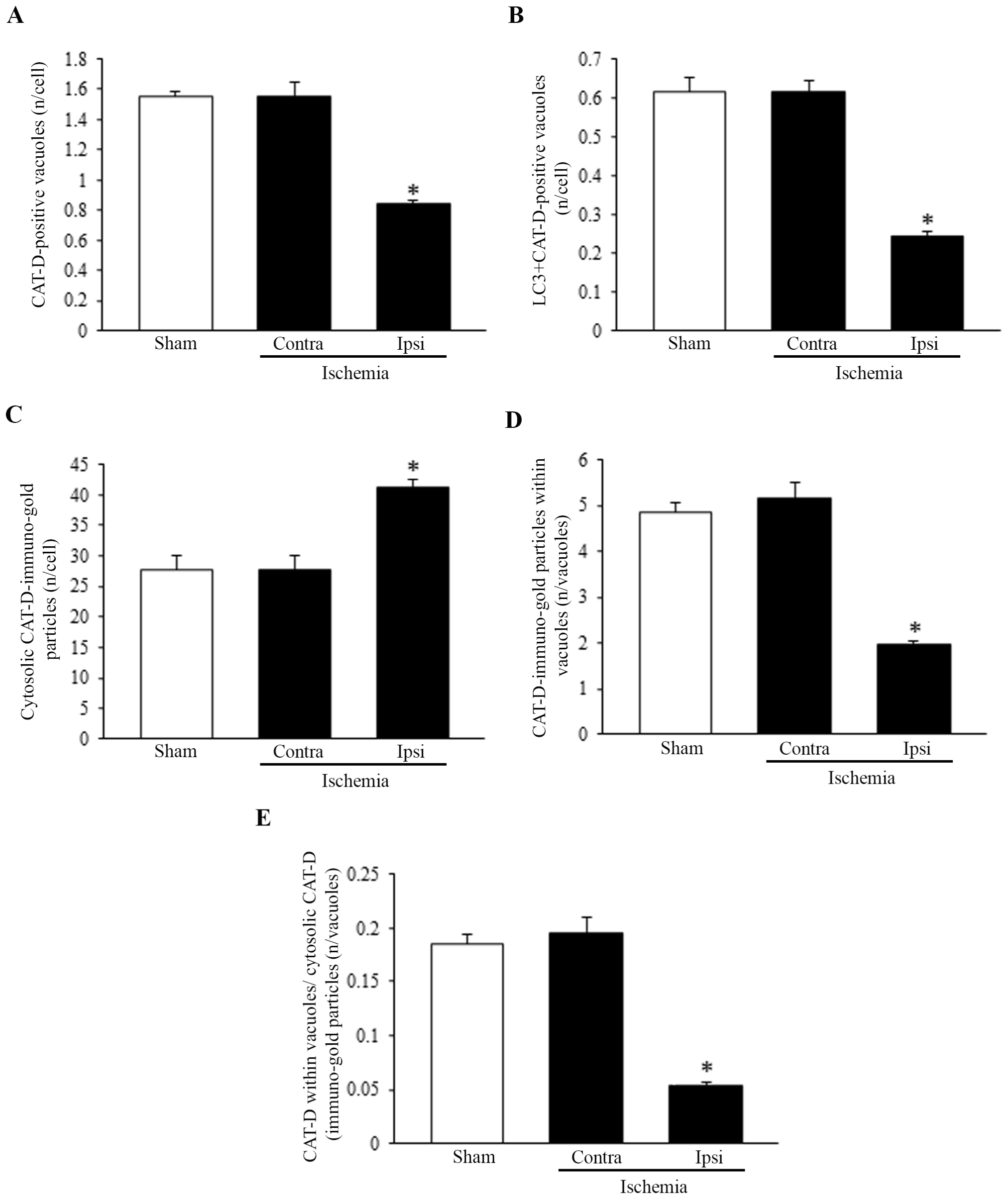

Figure S5. Within dorsal area penumbra cells cathepsin-D (CAT-D) molecules are reduced and dispersed from vacuoles concomitantly to a reduction in microtubule-associated protein I/II-Light Chain 3 (LC3)+Lysosomal associated Membrane Protein 1 (LAMP1) co-immuno-stained vacuoles.

Graph **A** reports the decrease in the number of CAT-D positive vacuoles per cell. Graph **B** reports the decreased in the number of LC3+CAT-D positive vacuoles per cell. Graph **C** reports an increased amount of CAT-D molecules counted in the cytosol per cells. Graph **D** reports a massive decrease in the amount CAT-D molecules counted within vacuoles. Graph **E** reports a decrease in the ratio between vacuolar and cytosolic amount of CAT-D molecules per cell. All graphs report the ultrastructural morphometry for CAT-D and LC3+CAT-D positive vacuoles from dorsal area penumbra (Ipsi/Ischemia) and the side contralateral to ischemia (Contra/Ischemia) and from sham-operated mice (Sham). Values are reported as the mean±S.E.M. per cell from a total of 120 cells for each group. *P≤0.05 compared with other groups.

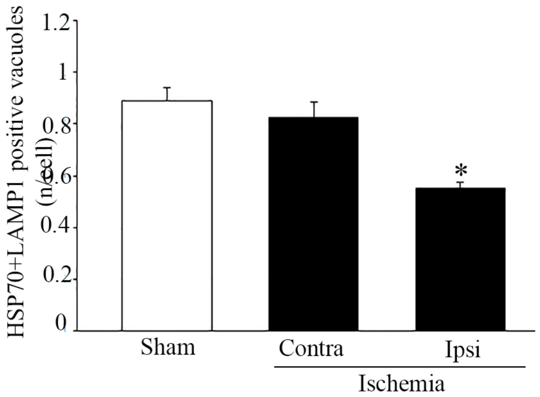

Figure S6. Heat Shock protein 70 (HSP70)+Lysosomal associated Membrane Protein 1 (LAMP1) co-immuno-stained vacuoles decrease within dorsal area penumbra.

Graph reports the number of HSP70+LAMP1 immuno-gold double-stained vacuoles per cell from area penumbra (Ipsi/Ischemia) and homologous regions from the contralateral side (Contra/Ischemia) and from sham-operated mice (Sham). Values are given as the mean±S.E.M. per cell from a total of 120 cells for each group. *P≤0.05 compared with other groups.
